# The potential of medicinal food plant *Panax ginseng* C. A. Mey. in managing chronic diseases via gut microbiota regulation: a systematic review of mechanisms and evidence

**DOI:** 10.3389/fphar.2025.1650565

**Published:** 2025-08-28

**Authors:** Meng Gao, Haijing Wang, Xiaojing Chen, Wensheng Wang, Yongmei Liu

**Affiliations:** ^1^ Neck-Shoulder and Lumbocrural Pain Hospital of Shandong First Medical University, Shandong First Medical University & Shandong Academy of Medical Sciences, Jinan, China; ^2^ Qingdao Traditional Chinese Medicine Hospital, Qingdao Hiser Hospital Affiliated of Qingdao University, Qingdao, China; ^3^ Qingdao Haici Traditional Chinese Medicine Medical Group North Campus(Qingdao Hongdao People’s Hospital), Qingdao, China; ^4^ Institute of Brain Science and Brain-Inspired Research, Shandong First Medical University and Shandong Academy of Medical Sciences, Jinan, Shandong, China

**Keywords:** medicinal food plant, Panax ginseng C. A. Mey., gut microbiota, pharmacology, plant metabolites, toxicology

## Abstract

Panax ginseng C.A. Mey. (PG), a well-documented medicinal food plant with generally recognized as safe status, exhibits therapeutic potential for managing metabolic disorders (type 2 diabetes mellitus (T2DM), obesity), inflammatory bowel diseases (IBD), and neurodegenerative conditions via modulation of the gut microbiota (GM). This systematic review of 102 studies reveals that ginsenosides (Rb1, Rg1, and Rg3) undergo biotransformation mediated by the GM into bioactive metabolites (e.g., compound K), enhancing their bioavailability by 3- to 5-fold (p < 0.01). Three core mechanisms were identified: 1) inhibition of the TLR4/NF-κB pathway reduces pro-inflammatory cytokine levels by 40%–60%; 2) upregulation of tight junction proteins (ZO-1/claudin-4) strengthens intestinal barrier function by 2.3-fold; and 3) selective GM modulation increases the relative abundance of probiotics (*Lactobacillus* ↑2.1-fold, Bifidobacterium ↑1.8-fold) while decreasing pathogenic bacteria (*Clostridium* ↓65%), collectively increasing short-chain fatty acid (SCFA) production by 3.2-fold, and activating AMPK/SIRT1 signaling. Clinical evidence supports PG’s efficacy: 15.2% reduction in fasting blood glucose levels in T2DM, 28.5% decrease in diamine oxidase (DAO) activity in IBD, and improvements in cognitive function scores (Mini-Mental State Examination scores increased by 2.4 points) in mild cognitive impairment. Emerging research further reveals a “microbiota-gut-brain axis” mediated by GM-derived metabolites acting via G protein-coupled receptors (GPCRs) and vagal pathways.

## 1 Introduction

Chronic diseases impose a substantial global health burden and cause high mortality rates (Cifuentes et al., 2025). The conventional “one molecule, one target” pharmacological paradigm, rooted in reductionist principles, exhibits intrinsic limitations in addressing the multifactorial pathogenesis of these complex disorders, underscoring the urgent need for innovative therapeutic strategies.

*Panax ginseng* C. A. Mey. (PG), a widely cultivated medicinal food plant (classified as a GRAS resource), is primarily harvested in autumn, washed, and processed via sun-drying or mechanical drying. As a cornerstone of traditional and modern integrative medicine, PG exerts pleiotropic therapeutic effects against chronic diseases through synergistic interactions with herbal preparations and dietary supplements. Its pharmacological activities, including glycemic control (Chen Q. et al., 2017), immune modulation (Qu et al., 2021), and antitumor efficacy (Wang et al., 2023), are primarily attributed to bioactive metabolites such as ginsenosides (e.g., Rb1, Rg1, Rg3, and Rh1) and polysaccharides. Recent studies have underscored the critical role of the GM in mediating the therapeutic effects of PG. Central to this mechanism is the GM’s facilitation of phytochemical biotransformation: it converts ginsenosides (e.g., Rb1→compound K (CK)), catechins, and quercetin into pharmacologically active metabolites, thereby enhancing both intestinal absorption and systemic bioavailability of PG-derived compounds (Santangelo et al., 2019; Furman et al., 2019). Comparative analyses of Asian and American ginseng chemotypes—defined by distinct chemical profiles—reveal variations in secondary metabolite composition (Liang et al., 2015) and tissue-specific accumulation patterns (Chen et al., 2015); these differences not only establish evidence-based quality control benchmarks for herbal preparations but also provide scientific rationale for their differential clinical applications (e.g., preferential use of Asian ginseng in metabolic disorders *versus* American ginseng in immune modulation). Notably, current evidence indicates that age-related dysbiosis (e.g., reduced microbial diversity, altered Firmicutes/Bacteroidetes ratios) or antibiotic-induced disruptions in GM homeostasis may significantly impair PG’s biotransformation efficiency and subsequent bioactivity (Chen Y. et al., 2017). This review aims to systematically synthesize PG’s multicomponent metabolites and their multitarget mechanisms in chronic disease management, highlight the GM’s role as a key determinant of PG’s bioactivity, evaluate its safety profile, and assess its therapeutic potential within integrative medicine frameworks.

The therapeutic effects of PG across diverse disease contexts are linked to GM modulation, though the robustness of supporting evidence varies notably. A comprehensive systematic review of the literature stratifies the mechanisms of ginsenosides into three evidence tiers: In diabetes management, for example, ginsenoside Rb1 enhances insulin sensitivity via GM-mediated metabolic transformation—a pathway validated by multi-omics profiling (Chen et al., 2022). This process involves augmenting probiotic abundance (e.g., *Lactobacillus* species), promoting SCFA biosynthesis, and suppressing the pro-inflammatory TLR4/NF-κB signaling axis. Beyond this well-supported mechanism, emerging evidence in antitumor research suggests ginsenosides Rg3 and CK may increase PD-1 inhibitor efficacy through GM modulation (Huang et al., 2022). However, these findings are currently limited to mouse models and lack clinical validation. Further complicating the landscape, speculative associations, such as the proposed “gut-brain axis” regulation in neuroprotection (Li et al., 2024), rely on indirect evidence where the GM composition changes correlate with behavioral improvement, but causal relationships remain unclear. Collectively, these findings underscore the need for further investigation to advance research in this field.

## 2 Methods and materials

*Panax ginseng* C.A. Mey. (Renshen; Ginseng Radix et Rhizoma) refers to the dried roots and rhizomes of plants in the *Panax* genus (family Araliaceae). Quality control adheres to HPLC analysis as specified in General Principle 0512 of the Chinese Pharmacopoeia. Each batch, when calculated on a dry weight basis, must contain no less than 0.30% combined ginsenosides Rg1 (C_42_H_72_O_14_) and Re (C_48_H_82_O_18_), and no less than 0.20% ginsenoside Rb1 (C_54_H_92_O_23_). Pharmacologically standardized batches further require ≥0.27% combined Rg1 and Re, and ≥0.18% Rb1 (Chinese Pharmacopoeia, 2025).

Physical characteristics of the product include round or nearly round thin slices prepared via moistening, cutting, and drying. Key morphological features are a grayish-yellow outer skin; pale yellowish-white to nearly white, powdery cross-sections with brownish-yellow vascular cambium rings; and a periderm displaying brown, dot-like resin ducts and radiating fissures. The slices have a lightweight and brittle texture, with a distinctive aroma and mildly bittersweet taste.

Compliance of PG (particularly wild or protected populations) with collection, processing, and trade regulations, including the Nagoya Protocol, the Convention on International Trade in Endangered Species of Wild Fauna and Flora (CITES), and plant quarantine requirements, requires verification. Product categories include: Raw materials/primary processed products: Dried ginseng roots (sliced or whole), ginseng powder, ginseng extracts (e.g., red ginseng extract). Finished products: Pharmaceuticals (e.g., ginseng capsules, tablets, and oral liquids); health supplements (e.g., ginseng tonics and energy drink additives); cosmetics (e.g., ginseng creams and essences).

Within our institution, a batch of herbal medicine sourced from Jilin Province is retained, bearing the batch number 2305210072.

This study employed a systematic and transparent literature search strategy aligned with the Population, Intervention, Comparison, Outcome, Study Design (PICOS) framework to ensure methodological rigor. The search targeted the following: 1) Population (chronic disease models/patients); 2) Intervention (ginseng/active plant metabolites); 3) Study Design (controlled settings); 4) Outcome (gut microbiota parameters); and 5) Study Types (clinical/preclinical research). Databases including PubMed, Web of Science, Embase, Cochrane Library, CNKI, Wanfang, and ClinicalTrials.gov were queried using a combination of keywords: interventions (“Panax ginseng,” “ginsenoside”), mechanisms (“gut microbiota,” “intestinal flora”), and diseases (“chronic disease,” “metabolic disorder”).

After removing duplicate articles with Zotero, two independent reviewers conducted title/abstract screening and full-text assessment based on predefined inclusion criteria (clinical or preclinical design, documented ginseng intervention, microbiota analysis, data integrity) and exclusion criteria (reviews, case reports, small sample sizes, and methodological limitations). Discrepancies in screening decisions were resolved through discussion with a third reviewer, with final judgments documented using a standardized form. Quality assessment of included studies was performed using the Cochrane Risk of Bias tool (for clinical studies) and SYRCLE’s Risk of Bias tool (for animal studies) to ensure the reliability of the evidence base.

**FIGURE 1:**
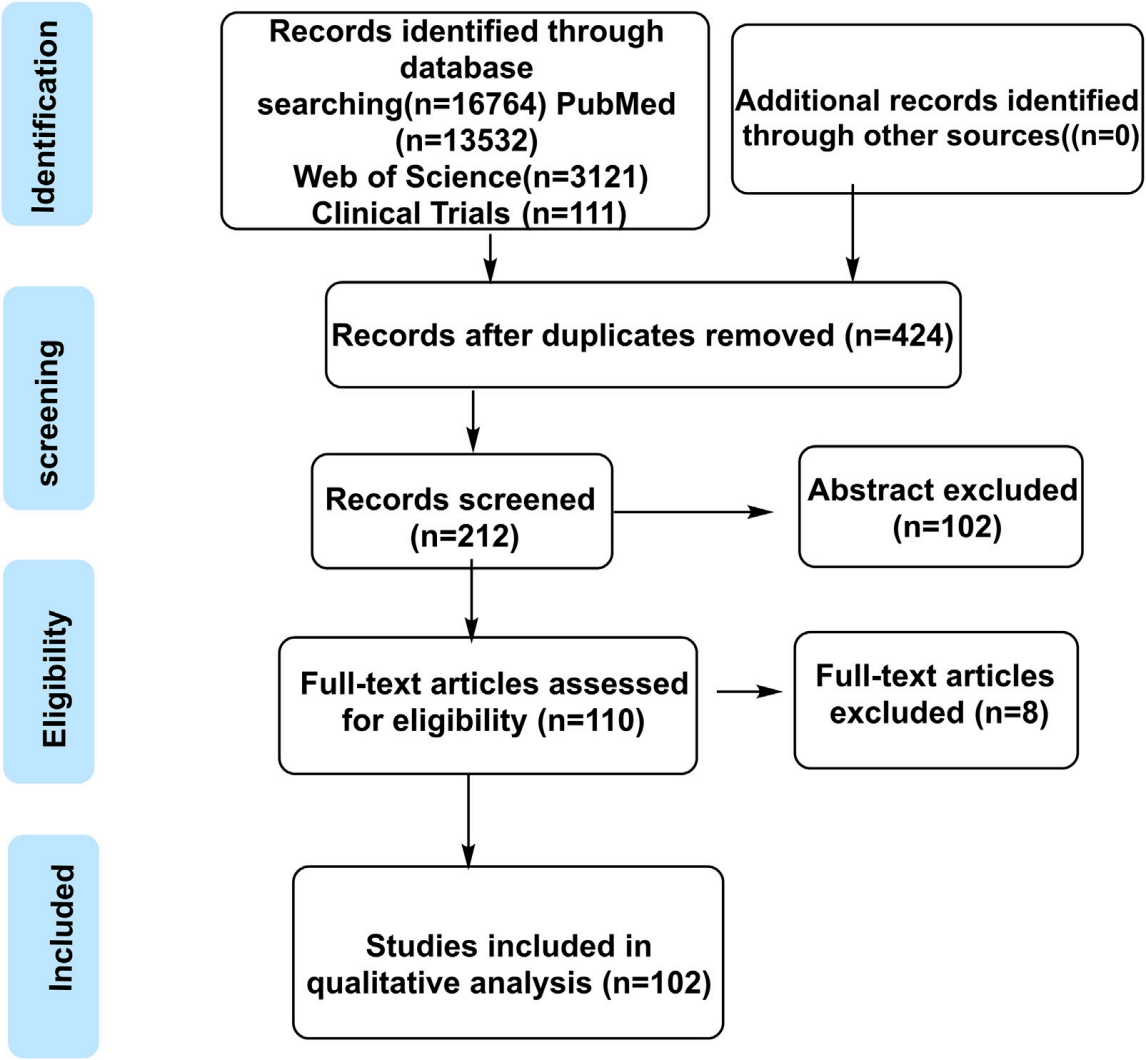
Flowchart of literature selection.

A total of 102 references were screened (final inclusion number to be updated by 2025). *Panax ginseng* C.A. Mey. (Latin name: *P. ginseng* C.A. Mey.), a perennial medicinal and edible plant, belongs to the genus *Panax* within the family Araliaceae. Taxonomic nomenclature and species illustrations were validated against authoritative sources, including Flora of China (1994) and The World Flora Online (http://www.worldfloraonline.org, accessed 10 March 2025), with chemical structure data sourced from PubChem and visualized using ChemDraw software. The literature retrieval flowchart is presented in Figure 1.

Methodological limitations of the current study are acknowledged as follows: 1) Most included studies did not explicitly describe the randomization procedure; 2) Only a minority adopted double-blind study designs; 3) Statistical power calculation was prespecified in few investigations; 4) Metagenomic sequencing was utilized in only a limited number of studies.

## 3 Gut microbiota and metabolism of plant metabolites of PG

PG, as a medicinal food plant, represents a highly dynamic research field. The gut microbiota, recognized for its pivotal role in host physiology, has been extensively acknowledged—particularly its function as a “superorganism” in conjunction with the host, mediating critical processes such as drug metabolism and other physiological functions (Zinöcker and Lindseth, 2018).

Long-term administration of PG extract (PE) induces significant modulation of GM composition: probiotics, including *Bifidobacterium*, *Lactobacillus*, *Allobaculum*, and *Clostridium*, are markedly upregulated, whereas pathogenic bacteria, such as *Butyricimonas*, *Parabacteroides*, *Helicobacter*, and *Alistipes*, are significantly downregulated (Qu et al., 2021). These findings demonstrate PE’s capacity to promote probiotic proliferation while suppressing pathogen growth, thereby providing foundational evidence for its regulatory effects on the GM structure in rat models (Sun et al., 2018).

The bioactivities of key PG-derived metabolites, including CK, ginsenosides Rg3, Rh2, 20(S)-protopanaxatriol (20(S)-PPT), and 20(S)-protopanaxadiol (20(S)-PPD), are critically dependent on GM-mediated metabolism. Ginsenosides generally exhibit poor gastrointestinal bioavailability but undergo extensive biotransformation mediated by the GM, with *in vivo* metabolic profiles aligning closely with *in vitro* GM fermentation patterns (Wang et al., 2023). Deglycosylated ginsenoside metabolites produced by the GM are then absorbed into the systemic circulation.

Notably, interindividual variability in GM composition results in divergent metabolic phenotypes. For example, ginsenoside Rb1 undergoes specific biotransformation by *Bifidobacterium spp*. within the GM to generate compound Rd (Dong et al., 2017). Oral administration of Rb1 and Re follows distinct metabolic pathways: Rb1/Re are metabolized via the GM through Rd to form 20(S)-PPD or CK, whereas Rg1 is processed through a distinct route involving retention of the Rg1 aglycone to generate 20(S)-PPT or Rh1 (Oh H. A. et al., 2015). PG plant metabolites further differentially regulate the GM across multiple taxonomic levels: At the phylum level, significant reductions in *Escherichia* abundance are observed; at the genus level, treatment with Rb1/Rb2/Rb3/Rc leads to decreased relative abundances of *Dorea*, *Prevotella*, and *Megasphaera*, concurrent with increased *Escherichia* (Zheng et al., 2021).

These structural and compositional changes enhance GM integrity and metabolic diversity. For example, GM hydrolysis plays a critical role in the appearance of CK in rat plasma following Rb1 administration (Akao et al., 1998). PE-induced modifications in GM composition, specifically the enrichment of Peptococcaceae, Rikenellaceae, and *Hungarian* legume-associated taxa, exhibit a strong positive correlation with increased absorption of ginsenosides (e.g., Rd and Rg3) (Kim et al., 2019). Exposure to stress models, including antimicrobial agents, fatigue, and acute cold, has been shown to suppress the production of deglycosylated ginsenoside Re metabolites, a phenomenon attributed to altered glycosidase activity in *Proteus*, *Lactobacillus*, and *Bacteroides* populations (Zhang et al., 2021). Ultra-high-performance liquid chromatography coupled with ion mobility-quadrupole time-of-flight mass spectrometry (UHPLC/IM-QTOF-MS) analysis of six biotransformation samples identified 248 ginsenoside/metabolite features, with desaccharification emerging as the dominant metabolic pathway. Notably, protopanaxatriol-type and oleanolic acid ginsenosides demonstrate heightened metabolic susceptibility (Mi et al., 2023). Probiotic supplementation synergizes with PG to optimize GM composition. In male Sprague–Dawley (SD) rats, probiotic-enriched feed increases the Rb1→CK conversion and gastrointestinal absorption of ginsenosides (Kim et al., 2019). Additionally, the PPD (protopanaxadiol) treatment group exhibits significant fecal microbiota alterations: increased relative abundances of *Proteus_9*, *Fusarium*, and *Monomonas* at the genus level and decreased *Bacteroides* and *Proteus* at the phylum level (Zhang et al., 2021).

**FIGURE 2:**
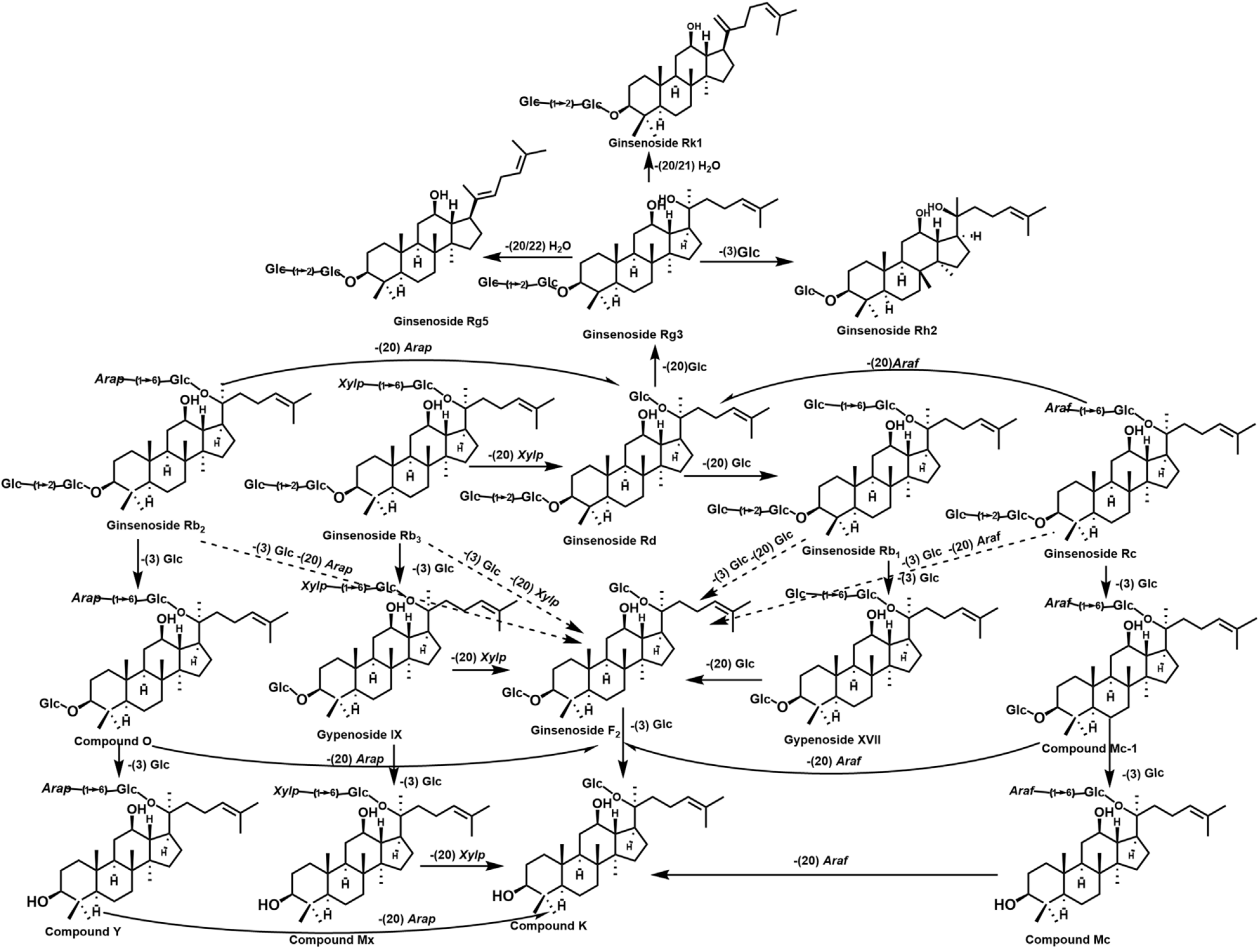
Plant metabolites in PG and their transformation in the human body. The figure shows the generation and conversion of different metabolites in PG, which are formed by the deglycosylation of sugar groups, including Rb1, Rb3, Rb2, Rc, and compound CK.

The dynamic interplay between PG plant metabolites and GM metabolism is summarized in Figure 2.

## 4 Pharmacodynamic material basis of PG in the treatment of diseases by regulating gut microbiota

### 4.1 The pharmacological effects of the plant metabolites in PG

PG, a highly valued traditional Chinese medicine (TCM), is often hailed as the “king of botanical drugs” due to its comprehensive pharmacological profile. Its primary plant-derived metabolites encompass three key categories: 1) Ginsenosides: Diverse analogs (e.g., Rb1, Rg1, Re); 2) Macronutrients: Carbohydrates and amino acids; 3) Micronutrients: Vitamins (B1, B2, C, etc.) and essential trace elements (potassium [K], sodium [Na], calcium [Ca], magnesium [Mg], etc.).

PG exerts multifaceted physiological effects, systematically summarized as follows: Immunomodulation: Ginsenosides and polysaccharides enhance immune competence by activating host defense mechanisms (Figure 2). Anti-fatigue and Anti-senescence: Improves physical performance and mitigates cellular aging processes. Endocrine Regulation: Stabilizes hormonal balance through systemic modulation of endocrine function. Neuroprotective Effects: Enhances cognitive function and memory retention via protection of the nervous system. Cardiovascular Protection: Improves myocardial function, increases cardiac output, and regulates glucose/lipid metabolism. Anticancer Activity: Inhibits carcinogenesis through metabolite-mediated cytostatic effects. Stress Adaptation: Enhances resilience to physical/psychological stressors while attenuating stress-induced damage.

**FIGURE 3:**
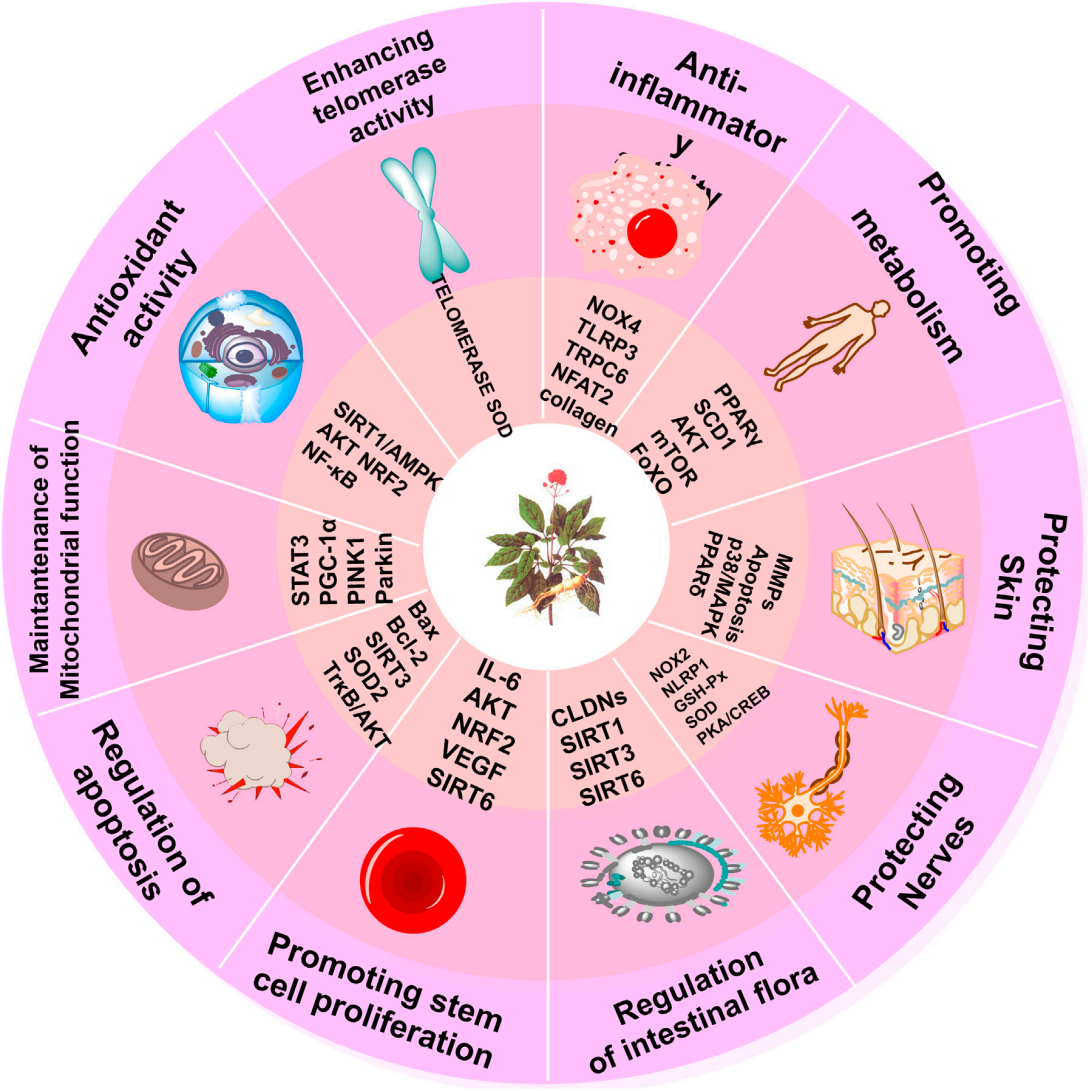
Pharmacological effects of PG. The figure shows the pharmacological mechanisms of action and targets of PG, including anti-inflammatory and antioxidant activity, promoting metabolism, protecting skin, protecting nerves, regulating GM, promoting stem cell proliferation, regulating apoptosis, maintaining mitochondrial function, and increasing telomerase activity.

The integrated pharmacological actions of PG are visually represented in Figure 3.

### 4.2 Metabolic disorders

#### 4.2.1 Diabetes

##### 4.2.1.1 Pathogenesis

Type 2 diabetes mellitus (T2DM) is characterized by progressive insulin resistance and pancreatic β-cell dysfunction, which collectively impair insulin secretion and glucose homeostasis, necessitating lifelong pharmacological management (Feng et al., 2018; Lao et al., 2014; Sharma et al., 2017). Contemporary chemical and biological research methodologies have enabled the discovery of novel therapeutic strategies for diabetes prevention and management of complications (Liu et al., 2022; Zhou et al., 2022). The pathogenesis of T2DM involves multiple interconnected mechanisms, including increased circulating free fatty acids and adipokines contributing to dysregulated lipid metabolism, pancreatic lipase hyperactivity, impaired dietary cholesterol absorption, gut microbiota dysbiosis, central appetite dysregulation, nuclear receptor (e.g., PPAR and LXR) signaling abnormalities, and dysfunctional AMP-activated protein kinase (AMPK) pathways and adipose tissue homeostasis.

##### 4.2.1.2 Plant metabolites for treating diabetes in PG

Ginsenoside Rb1 exerts multifaceted anti-diabetic effects through its potential prebiotic properties, which regulate specific intestinal microbiota and their metabolites, which are key contributors to diabetes-related metabolic disorders and insulin resistance. In diabetic Kkay mice, Rb1 significantly reduces blood glucose levels, improves oral glucose tolerance test (OGTT) performance, decreases serum insulin levels and homeostasis model assessment of insulin resistance (HOMA-IR) index, lowers hepatic index, alleviates pancreatic injury, reverses GM dysbiosis, and modulates fecal free fatty acid profiles (Zhou et al., 2023). Similarly, Rg1 demonstrates multifaceted anti-diabetic activities: in high-fat diet (HFD)- and streptozotocin (STZ)-induced T2DM rats, it significantly reduces blood glucose levels, improves insulin sensitivity, attenuates oxidative stress and inflammation, normalizes blood lipid profiles, and positively modulates intestinal microbial composition (Peng et al., 2022). As the primary circulating metabolite of PG in serum, CK not only confers renal protection but also modulates GM composition and downregulates the expression of TLR4 signaling pathway proteins induced by imidazole propionate (Chen et al., 2022). In male Wistar rats, ginsenoside Rb1 exhibits significant hypoglycemic activity via a specific metabolic pathway (Rb1→Rd→F2→CK), where the bioconversion rate of CK increases from 14.0% to 86.7% following 8 h of incubation. Ginseng pectin (GPS) synergizes with Rb1 to restore GM homeostasis and increase fecal D-glucosidase activity, thereby exerting potent anti-diabetic effects (Li et al., 2018). GPS treatment further ameliorates hyperglycemia, hyperlipidemia, and hepatic steatosis in T2DM rats by activating AMP-activated protein kinase (AMPK) and promoting the phosphorylation of acetyl-CoA carboxylase, which subsequently reduces the expression of sterol regulatory element binding protein-1c (SREBP-1c) and fatty acid synthase (FAS) (Ren et al., 2023a). Additionally, 25-hydroxy-protopanaxatriol (T19), a novel ginsenoside derivative derived from PG, inhibits α-glucosidase and protein tyrosine phosphatase 1B (PTP-1B) *in vitro*. These inhibitory effects, combined with T19’s ability to modulate glucose and lipid metabolism via beneficial bacterial interactions, effectively reduce blood glucose and lipid levels, increase insulin sensitivity, and ameliorate hepatic and pancreatic histopathology (Xu et al., 2020).

##### 4.2.1.3 Therapeutic mechanism

**FIGURE 4:**
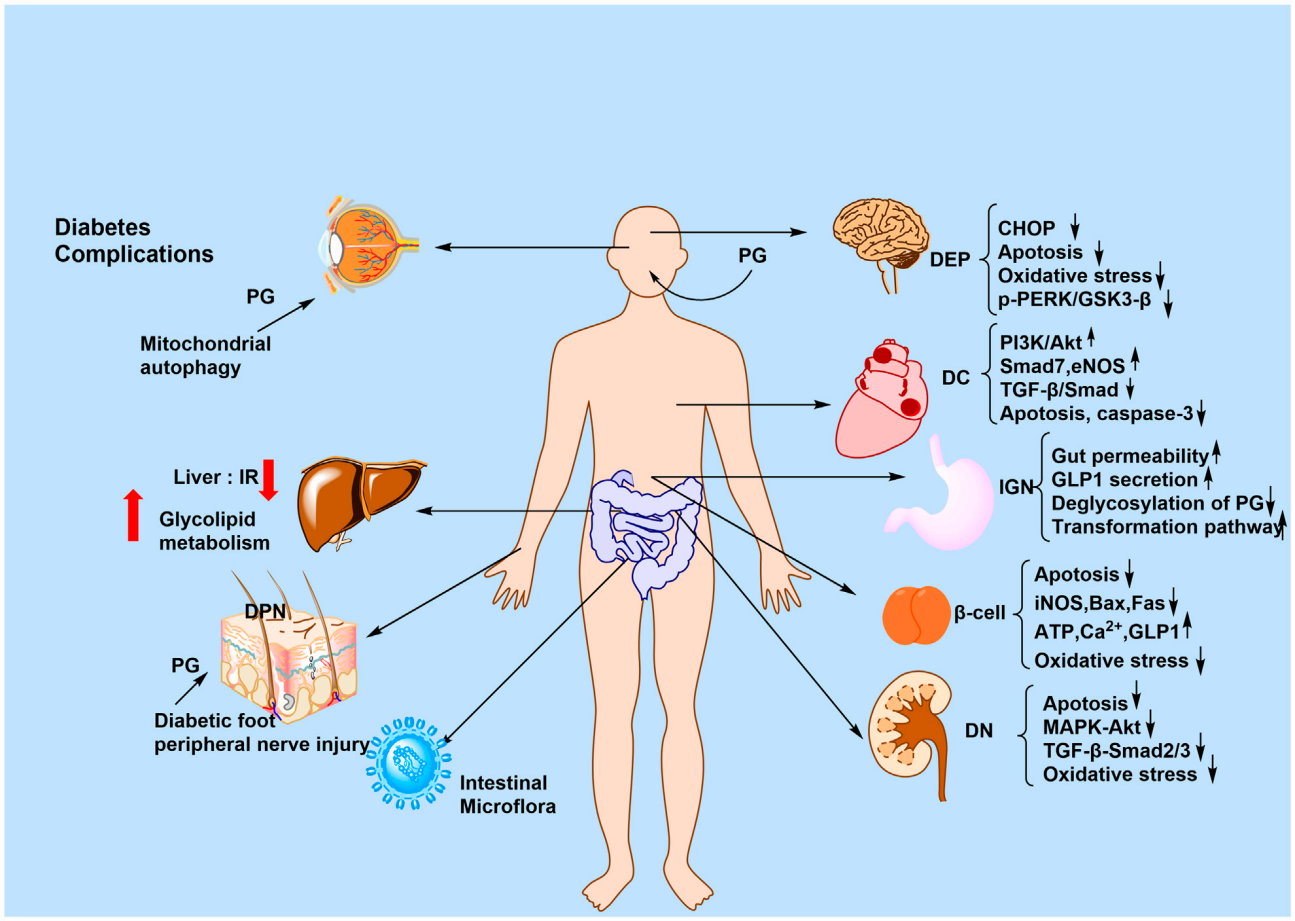
Anti-T2DM mechanism of PG. The figure shows the mechanism of action of PG for the treatment of T2DM and its complications, summarized in terms of the brain, heart, stomach, kidney, liver, skin, and intestines. The signaling pathways involved may include p-PERK/GSK3-β, PI3K/AKT, and MAPK/AKT.

The active plant metabolites of PG, including Rb1, Rg1, CK, and T19, exert anti-diabetic effects through multiple synergistic mechanisms: (1) modulation of the gut microbiota; (2) mitigation of metabolic disorders; (3) activation of the AMPK signaling pathway; (4) exertion of anti-inflammatory and antioxidant effects; and (5) protection of organ functions (e.g., pancreas and kidneys). Collectively, these effects are mediated via the gut microbiota–metabolism–immunity network. The anti-T2DM mechanism of PG is summarized in Figure 4.

#### 4.2.2 Obesity

##### 4.2.2.1 Pathogenesis

Obesity remains a major global public health concern (Safaei et al., 2021). Numerous obesity-associated chronic diseases, including T2DM, cardiovascular disease, and non-alcoholic fatty liver disease (NAFLD), represent leading causes of global mortality (Hu et al., 2017).

##### 4.2.2.2 The plant metabolites for treating obesity in PG

PG exerts anti-adipose effects by inhibiting peroxisome proliferator-activated receptors γ/α (PPAR-γ/PPAR-α). Additionally, PG modulates EBP-α, potentially augmenting lipid oxidation and energy expenditure. Pharmacological interventions utilizing PG have shown significant lipid-regulatory effects mediated by GM modulation. In HFD-induced obese rats, PG treatment alleviates obesity symptoms, increases fecal bile acid excretion, and increases gut microbiota (GM) diversity alongside the relative abundance of *Prevotella_9* (Zhao et al., 2021). Mechanistic investigations in db/db mice further reveal that PG-derived *gut enterococci* (*Enterococcus faecalis*) convert dietary plant metabolites into unsaturated long-chain fatty acids (LCFAs), notably myristic acid (MA), via acyl-CoA thioesterase (ACOT)-mediated biosynthesis. Intriguingly, PG concurrently downregulates MA production through as-yet-uncharacterized mechanisms (Quan et al., 2020).

In dyslipidemic rat models, PE enriches the abundance of beneficial bacterial taxa (*Akkermansia, Bifidobacterium, Bacteroides,* and *Proteus*) and increases SCFA biosynthesis (acetate, propionate, butyrate, and valerate). These changes improve lipid metabolism through GM remodeling and activation of the AMPK signaling pathway (Ren et al., 2023b). Ginsenosides, including Rb1, Rb2, Rb3, Re, Rg1, Rg3, Rh2, Rh4, and F2, exert comprehensive cardiometabolic protective effects by increasing antioxidant enzyme activities, promoting fatty acid β-oxidation and autophagy, and modulating the GM to mitigate oxidative stress (Jin et al., 2023).

Notably, PPD maintains intestinal barrier integrity in leptin-deficient (ob/ob) mice exhibiting Parkinson’s disease-like pathologies. However, specific bacterial taxa (e.g., *Lactobacillus* spp.) that mediate this effect require further characterization (Lv et al., 2022).

##### 4.2.2.3 Therapeutic mechanism

**FIGURE 5:**
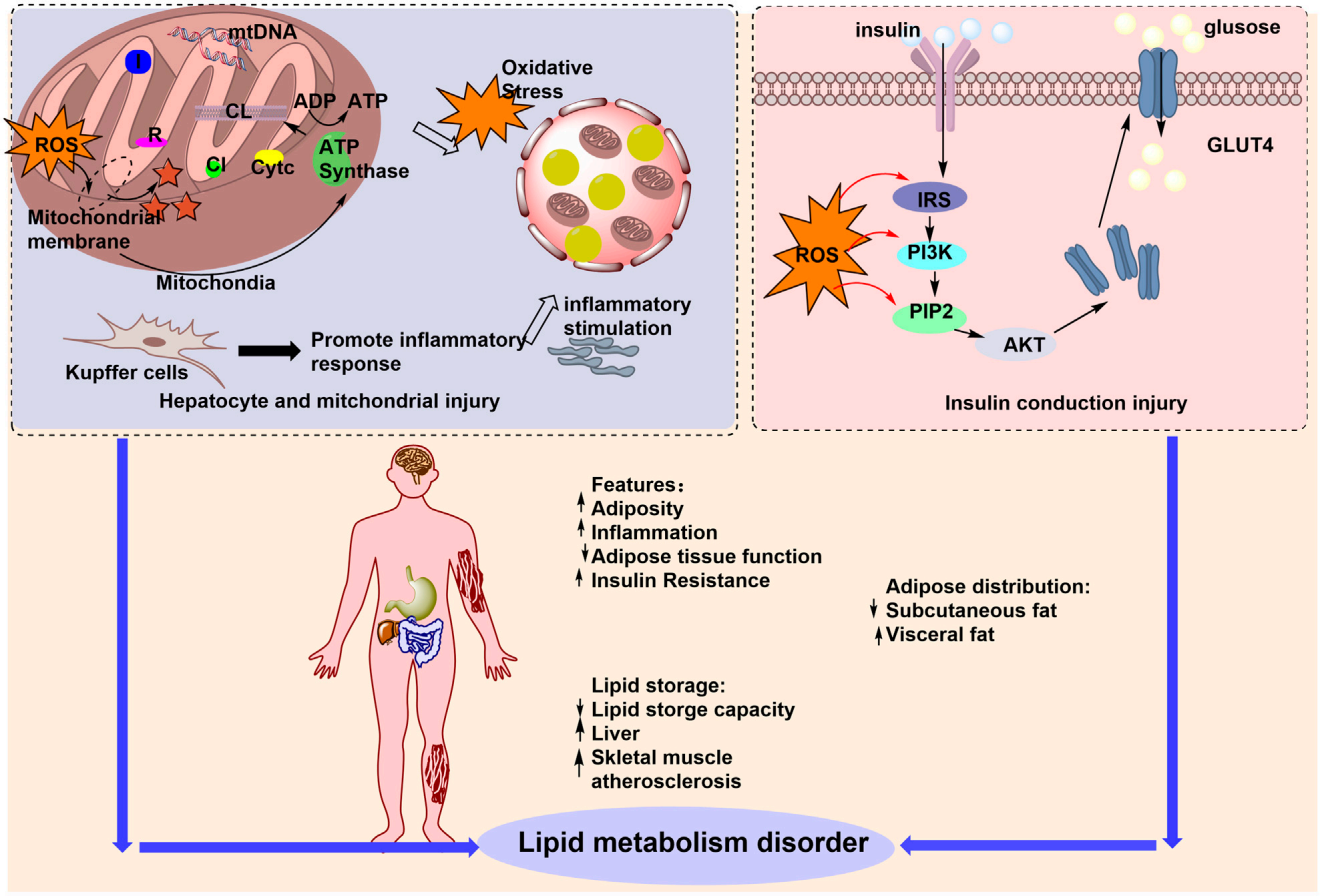
PG regulation of lipid metabolism by the GM for the treatment of obesity. The figure illustrates the multiple factors contributing to lipid metabolism disorders that lead to obesity, including increased adiposity, upregulation of inflammation, and increased lipid retention capacity. PG can improve lipid metabolism disorders by regulating ROS, affecting the GM, and treating obesity.

PG combats obesity through multitarget mechanisms, including 1) modulation of GM; 2) increasing lipid oxidation via PPAR-γ/PPAR-α inhibition and AMPK activation; 3) promotion of SCFA production (acetate, propionate, butyrate); 4) reduction of oxidative stress and inflammation; and 5) improvement of intestinal barrier integrity. Key plant metabolites, such as ginsenosides Rb1 and Rg3, further regulate bile acid excretion and inhibit myristic acid synthesis, exerting synergistic anti-obesity effects through the gut–liver–metabolic axis. The mechanisms by which PG regulates lipid metabolism via the GM are summarized in Figure 5.

### 4.3 Gastrointestinal and liver diseases

#### 4.3.1 Colitis

##### 4.3.1.1 Pathogenesis

Colitis, a chronic inflammatory disorder that promotes a tumor-prone microenvironment and ranks as the third leading cause of cancer-related mortality globally (Bocchetti et al., 2021), is primarily driven by GM dysbiosis among multiple contributing factors. Such dysbiosis disrupts intestinal barrier integrity, impairs mucosal immune function, and triggers excessive production of pro-inflammatory cytokines, ultimately leading to mucosal erosion, ulceration, and the development of clinically relevant IBDs. The spectrum of IBDs encompasses not only IBD itself but also irritable bowel syndrome, intestinal ischemia–reperfusion injury, necrotizing enterocolitis, radiation proctitis, and NSAID-induced enteritis. Notably, ginsenosides counteract these pathological processes through multifaceted mechanisms: attenuating intestinal inflammation, promoting intestinal barrier repair, maintaining GM diversity, and reducing the risk of colitis-associated colon cancer.

##### 4.3.1.2 Plant metabolites for treating colitis in PG

In Caco-2 cell models, co-treatment with colitis-derived intestinal microorganism citrate synthase (CS) and PG + UC (PG under ulcerative colitis conditions) significantly impaired P-glycoprotein (P-gp) function. Concurrently, PG directly disrupted zonula occludens-1 (ZO-1) localization and suppressed tight junction (TJ) protein expression (Yang et al., 2021). In dextran sulfate sodium salt (DSS)-induced colitis models, PG treatment partially reversed the DSS-induced reduction in β-glucosidase activity, alleviated colitis symptoms, and enhanced systemic Rb1 exposure through two mechanisms: promoting microbial deglycosylation of Rb1 and facilitating its absorption by intestinal epithelial cells (Shen et al., 2018).

Ginsenoside Rg1 exerts immunomodulatory effects by increasing secretory immunoglobulin A (SIgA) secretion and modulating the expression of interleukin (IL)-2, IL-4, IL-10, and interferon-γ (IFN-γ). Concurrently, Rg1 regulates GM composition. Metabolites generated by Rg1 through metabolism mediated by Lachnospiraceae family bacteria further increase the proliferation of T cells and regulatory T cells (Tregs), thereby ameliorating colonic inflammation and restoring mucosal barrier integrity (Yousuf et al., 2022).

Similarly, ginsenoside Rh4 alleviated antibiotic-induced pathophysiological changes, intestinal barrier disruption, and inflammation in mice, concomitant with beneficial shifts in GM diversity and composition, thereby underscoring its bidirectional interaction with host microbiota (Bai et al., 2021a). Rk3 treatment mitigated HFD-induced intestinal barrier dysfunction in mice by upregulating tight junction proteins (ZO-1, claudin, and occludin), reducing colitis-associated cytokines, oxidative stress, and macrophage infiltration, while concurrently inducing favorable GM modulation (Chen et al., 2021; Bai et al., 2021b).

Comparative *in vitro* studies demonstrated that red ginseng (RG) and betel nut exhibit prebiotic effects, with RG showing superior efficacy to betel nut in improving GM structure and alleviating UC symptoms in TNBS-induced UC rat models (Wan et al., 2024). Mechanistically, PG restores mTOR-dependent autophagy and modulates GM composition (Wang et al., 2021).

##### 4.3.1.3 Therapeutic mechanism

PG and its bioactive metabolites, including Rg1, Rh4, Rk3, and red ginseng, exert anti-colitis effects through multiple synergistic mechanisms: 1) Enhancing intestinal barrier integrity by upregulating tight junction proteins (ZO-1, claudin, and occludin) and restoring β-glucosidase activity; 2) modulating GM composition by increasing the relative abundance of beneficial taxa (*Lactobacillus and Bifidobacterium*) and suppressing pathogenic bacteria; 3) Regulating immune homeostasis via increasing regulatory Tregs and SIgA, coupled with cytokine balancing (reducing pro-inflammatory IL-2, IL-4, IL-10, and IFN-γ); 4) Activating protective signaling pathways, such as mTOR-dependent autophagy; and 5) Promoting Rb1 bioavailability through microbial metabolism. Collectively, these coordinated actions underscore ginseng’s critical role in restoring intestinal homeostasis during colitis alleviation.

#### 4.3.2 Liver diseases

##### 4.3.2.1 Pathogenesis

The liver plays vital physiological roles in synthesizing plasma proteins, secreting bile acids, metabolizing xenobiotics, and regulating glucose and lipid homeostasis. Dysregulated metabolism of these critical substances can give rise to diverse hepatic disorders and their associated complications, contributing to significant global morbidity and mortality. These disruptions pose substantial public health challenges and economic burdens (Marchesi et al., 2016).

##### 4.3.2.2 The plant metabolites for treating liver diseases in PG

Ginsenoside Rg1 enhances intestinal barrier function by upregulating tight junction protein levels and secretory SIgA, while mitigating alcohol-induced inflammatory responses through inhibition of the TLR4/NF-κB signaling pathway. Additionally, Rg1 modulates GM composition: *Verrucomicrobia, Bacteroidetes, Akkermansia, Bacteroidae,* the Lachnospiraceae_NK4A136_group, and *Alloprevotella* exhibit positive correlations with intestinal barrier indices and negative correlations with liver inflammation indices (Xia et al., 2022). Analyses of cecal microbial diversity revealed that alcohol exposure significantly increased the abundance of *Bacteroides* S24-7 and *Proteus* S24-7, while reducing the relative abundances of SCFA-producing bacteria, including *Akkermansia* (phylum Verrucomicrobia), *Allobaculum, Luminococcus*, and *Adlercreutzia* (phylum Actinobacteria). Notably, fermented ginseng (FG) effectively reverses these alcohol-induced alterations in gut microbiota composition (Fan et al., 2019).

PPD ameliorates dysregulated glucose and lipid metabolism while improving liver function. Strong correlations were observed between the GM and anti-inflammatory/antioxidant metabolites in mice. Furthermore, (20R)-PPD enhances the integrity of the intestinal wall. Concurrently, downregulation of TNF-α, malondialdehyde (MDA), and superoxide dismutase (SOD) expression confirms PPD’s antioxidant and anti-inflammatory effects (Fang et al., 2023; Feng et al., 2023).

##### 4.3.2.3 Therapeutic mechanism

PG alleviates liver diseases through multifaceted mechanisms. First, via the gut–liver axis, ginsenoside Rg1 and FG restore intestinal barrier integrity and rebalance gut microbiota composition, thereby reducing endotoxin translocation. Second, PG exerts anti-inflammatory and antioxidant effects to mitigate hepatic damage. Third, metabolic regulation is achieved through PPD, which enhances glucose and lipid metabolism while improving liver function via mannose-derived metabolites such as SCFA. Fourth, FG promotes microbial detoxification by antagonizing alcohol-induced gut dysbiosis and enriching liver-protective bacterial populations. Collectively, these synergistic actions alleviate liver inflammation, oxidative stress, and metabolic dysfunction.

#### 4.3.3 Diarrhea

Diarrhea, a leading global cause of morbidity and mortality, manifests as an acute infectious condition (Shankar and Rosenbaum, 2020) and can induce severe complications, including hypovolemia, electrolyte imbalance, malnutrition, and cutaneous damage, particularly when prolonged (Kyu et al., 2025). Its pathogenesis involves multifactorial drivers such as bile acid metabolism disorders, viral infections, and *Escherichia coli* colonization, with emerging evidence highlighting GM dysbiosis as a critical factor (Fan and Pedersen, 2021). Notably, restoring GM homeostasis has demonstrated both preventive and therapeutic potential against diarrhea (Gallo et al., 2016).

FG demonstrates notable therapeutic efficacy in antibiotic-induced GM dysbiosis models, as evidenced by its ability to restore GM structure in antibiotic-treated rats (with dose-dependent effects on microbial composition) (Qu et al., 2021). In male SD rats, FG alleviates diarrhea and colitis symptoms while normalizing colonic expression of immune factors (TNF-α, IL-1β, IL-6, and IL-10) (Li et al., 2019), and further downregulates pro-inflammatory cytokines to restore GM homeostasis. Comparative investigations with water-extracted ginseng (WGP) have revealed distinct mechanisms underlying antibiotic-associated diarrhea: WGP significantly modulates GM composition and diversity, particularly through the enrichment of *Lactobacillus* populations as key responders, and achieves GM rebalancing via dual mechanisms: structural restoration and modulation of metabolic processes (Li et al., 2019).

### 4.4 Cancer

#### 4.4.1 The anticancer mechanism of PG

**FIGURE 6:**
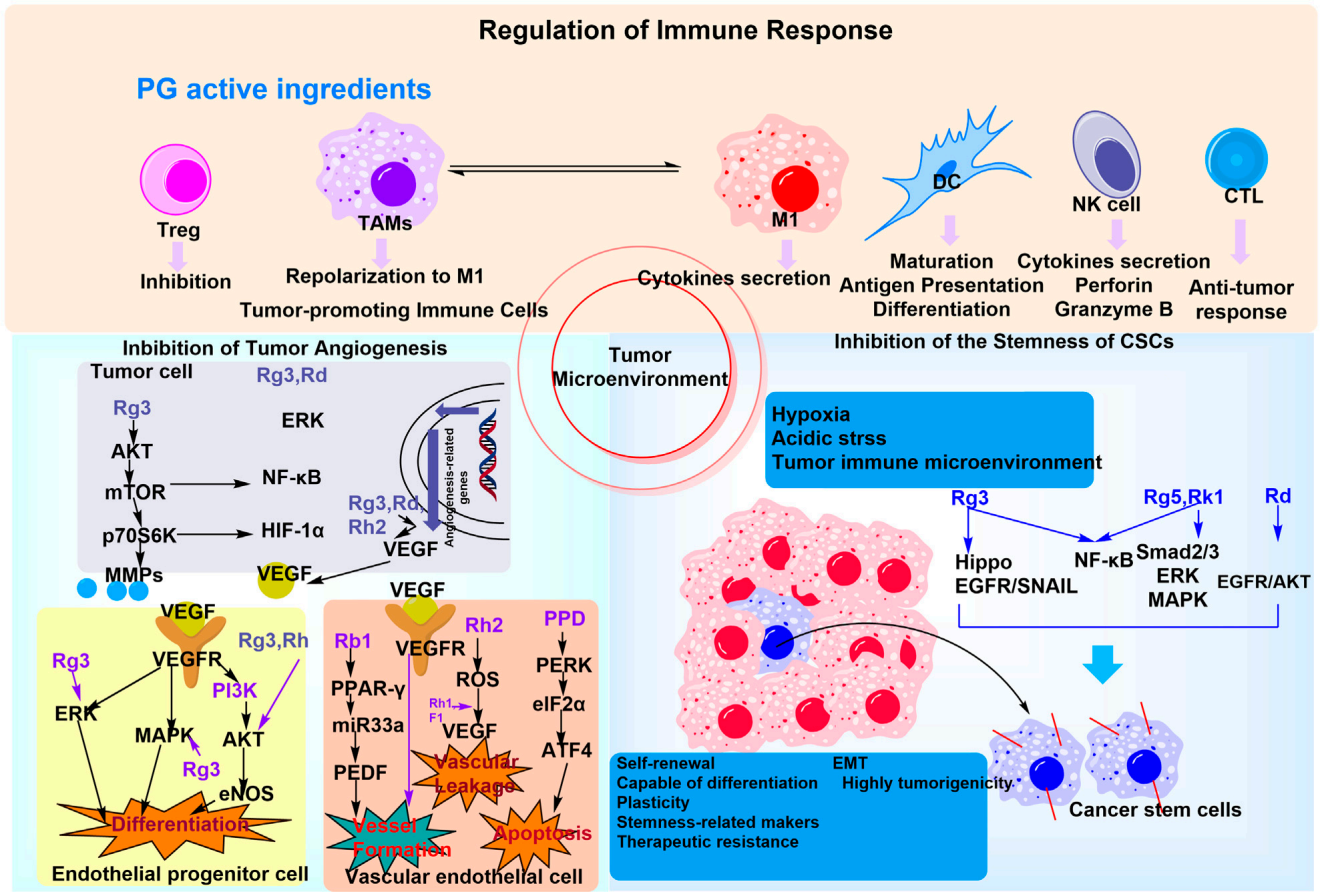
The mechanism of PG plant metabolites regulating the tumor microenvironment and anti-inflammation and immune regulation through the GM. PG may modulate immune cells to improve the GM by regulating the tumor immune microenvironment, in addition to involving multiple signaling pathways to promote tumor cell apoptosis.

PG, a medicinal plant endemic to Asian countries with a millennia-long history of traditional use, has garnered significant attention for its pharmacological properties. Ginsenosides, a class of bioactive metabolites derived from PG, exhibit promising anticancer potential, with multifaceted effects on the tumor microenvironment (TME). For instance, Rg3, Rd, and Rk3 have been shown to inhibit tumor angiogenesis; Rg3, Rh2, and M4 modulate immune cell function; and Rg3, Rd, and Rg5 suppress the dormancy of tumor stem cells. GP, including acidic polysaccharides, and those extracted from PG fruits and leaves, principally regulate the TME through immune cell stimulation (Li et al., 2021). The antitumor effects of specific ginsenosides, including Rg3, Rh2, Rg5, and CK, are well-documented, with proposed mechanisms involving apoptosis induction, immune response enhancement, chemotherapy resistance reversal, and regulation of key signaling pathways such as MAPK, PI3K/Akt/mTOR, Wnt/β-catenin, NF-κB, ASK-1/JNK, AMPK, and EGFR/Akt/SOX2. Notably, the PI3K-AKT/NF-κB signaling pathway serves as a critical mediator through which ginsenoside Rb1 and CK exert their anti-gastric cancer effects (Wang S. et al., 2020). The role of PG-derived plant metabolites in regulating the TME via the GM is schematically illustrated in Figure 6.

#### 4.4.2 The plant metabolites in PG for treating cancer

CK, but not Rb1, significantly inhibited the proliferation of HGC-27 gastric cancer cells by downregulating cyclins B1 and D1, suppressing the anti-apoptotic protein Bcl-2, and activating pro-apoptotic proteins Bax and Caspase-3 through inhibition of the PI3K/AKT/NF-κB signaling pathway (Wan et al., 2022). This differential effect underscores the role of GM-mediated regulation of PG metabolites in clinical applications.

Combination therapy with GPs and αPD-1 monoclonal antibody potentiated antitumor responses by increasing valeric acid production and reducing L-kynurenine levels, thereby decreasing the kynurenine/tryptophan (Kyn/Trp) ratio to suppress regulatory Tregs and promote expansion of effector T cells (Huang et al., 2022). Mechanistically, GPs modulated GM composition and regulated tryptophan metabolism to drive these effects.

GP-n (ginseng crude polysaccharide) exhibited anti-melanoma activity through mechanisms involving *Bifidobacterium* enrichment and modulation of tumor-associated SCFAs. Specifically, *Allobaculum* and *Bifidobacterium* displayed significant negative correlations with tumor weight and positive associations with SCFA levels (Xie et al., 2023). In qi-deficient BALB/c nude mice bearing liver cancer, treatment with PG suppressed tumor growth by remodeling fecal metabolites and altering GM composition (Hou et al., 2022).

### 4.5 Other diseases

Numerous studies have demonstrated that PG, ginsenosides, and GPs exert potential therapeutic effects on various other diseases. In male Wistar rats with spleen deficiency syndrome, co-administration of PG and wild jujube upregulated the relative abundances of *Firmicutes, Bacteroidetes, Lactobacillus*, and *Bifidobacterium*, while reducing the relative abundances of *Actinobacteria, Proteobacteria, Streptococcus, Escherichia, Shigella, Veillonella,* and *Enterococcus*. This modulation reversed the pathological state of GM imbalance associated with spleen deficiency (Li et al., 2022).

#### 4.5.1 Immunity and inflammatory diseases

Intragastric administration of ginsenoside F2 was found to ameliorate atopic dermatitis (AD)-like cutaneous symptoms, reduce inflammatory cell infiltration, and suppress intestinal and cutaneous inflammatory responses in AD mice via the GPCR/NF-κB signaling pathway, thereby improving skin AD symptoms. These findings first revealed the mechanism by which ginsenoside F2 alleviates AD through the propionic acid (PA) (a GM-derived metabolite)-intestinal–skin axis (Li et al., 2024). Treatment with ginsenoside Rg decreased interleukin (IL)-4, IL-5, and IL-13 levels in the colon and restored the relative abundances of *Bacteroides* and *Actinomycetes*, which are populations suppressed by *ovalbumin* and *Firmicutes* (a bacterial phylum) induced by ovalbumin in the GM. Additionally, ginsenoside Rd effectively alleviated ovalbumin-induced allergic rhinitis in mice (Kim et al., 2015).

#### 4.5.2 Aging and neuroprotection

Intragastric administration of ginsenoside Rb1 ameliorated cellular senescence phenotypes in C57BL/6 mice by reducing senescent cell accumulation and upregulating the expression of tight junction proteins CLDN2, CLDN3, CLDN7, and CLDN15 in jejunal tissues (Lei et al., 2022). Pharmacokinetic investigations further revealed that stressed and antimicrobial-treated rats exhibited altered systemic exposure to Rb1, potentially mediated by changes in fecal excretion kinetics (Kang et al., 2016).

Ginsenoside Rb1 exerted protective effects against morphine-induced conditioned place preference (CPP) in mice. Concurrently, Bovis callosus and ginsenoside Rg1 mitigated morphine dependence through inhibition of tryptophan metabolism, reduction of 5-hydroxytryptamine (5-HT) levels, and downregulation of 5-HT receptor subtypes 1B and 2A (5-HTR1B/5-HTR2A) expression (Chen et al., 2022).

Comparative studies of PG preparations demonstrated that garden ginseng (GG) and forest ginseng (GF) mitigated aging-associated apoptosis and inflammation in rats through regulation of the PI3K/AKT/mTOR and SIRT1/NF-κB signaling pathways, restored GM diversity, and enriched beneficial bacterial taxa such as *Lactobacillus*. FG exhibited superior anti-aging efficacy by additionally targeting oxidative stress and the microbe–intestine–brain axis (Hao et al., 2022).

In *Caenorhabditis elegans* models, PG treatment extended lifespan, enhanced gut microbiota composition, and increased the relative abundance of beneficial bacteria. Non-targeted metabolomic analysis further revealed five key pathways underlying PG’s anti-aging effects: neuroactive ligand–receptor interaction, tricarboxylic acid (TCA) cycle, pyruvate metabolism, ascorbic acid/uronic acid metabolism, and D-arginine/D-ornithine metabolism (Xu et al., 2023).

#### 4.5.3 Metabolism and cardiovascular diseases

Ginsenoside-treated rat models, particularly those treated with Rb1, show that the ginsenoside treatment exerts beneficial effects on blood pressure regulation and the formation of intracranial aneurysms. GM analysis revealed that ginsenoside treatment significantly modulated the relative abundances of several bacterial genera, including *Clostridium*, *Rose incense*, *Rumen cocci*, and *Spirochetes*. Additionally, several key metabolites that regulate blood pressure, such as benzoic acid, N-acetyl-5-hydroxytryptamine, prostaglandin F2α, and vitamin D2, were identified (Zeng et al., 2023). PG can increase metabolism associated with spleen qi deficiency syndrome, and its metabolites, along with UPE intensity, hold potential as biomarkers for the diagnosis and monitoring of this syndrome (Wang N. et al., 2020).

#### 4.5.4 Endocrine regulation

PG, RG, and black ginseng (BG) may exert therapeutic effects on hyperthyroidism through regulation of the hypothalamus–pituitary–thyroid (HPT) axis. Additionally, steroids of the hypothalamus-pituitary-adrenal (HPA) axis may interact with the HPT axis to collectively influence thyroid function.

#### 4.5.5 Mechanism of intestinal microbiota remodeling

Sugars and ginsenosides in water extract of ginseng (WEG) act as energy sources for specific GM, positively regulating GM composition and ultimately reshaping the intestinal microbial ecosystem, thereby activating multiple molecular and cellular signaling pathways (Zhou et al., 2021). PG regulated propionic acid levels more effectively than other groups. Additionally, PG increases the relative abundance of beneficial bacteria and reduces the relative abundance of harmful bacteria in rats with spleen qi deficiency syndrome (Zhang M. et al., 2023; Wang et al., 2023). Ginsenosides derived from stems and leaves improve the composition of GM, particularly increasing the abundance of probiotics (Su et al., 2023).

Administration of PE altered GM structure, resulting in reduced TM7 abundance and increased abundances of *Proteus*, *Methylbacillaceae*, *Parasitobacterium*, and *Sauteria*. Following long-term PE treatment, levels of IL-4, IL-10, and SIgA were significantly increased, showing strong positive correlations with *Bifidobacterium* and *Lactobacillus* abundances. These findings demonstrate that long-term PE administration exerts beneficial effects on host intestinal metabolism, immune function, anti-inflammatory processes, and GM structure (Sun et al., 2018). The pharmacological effects of the PG plant metabolites are shown in Table 1.

**TABLE 1:** Pharmacological activities of the plant metabolites of PG.

metabolite(s)/Extract(s)	Models	Dose	Detailed activities/mechanisms of action	Application	References
Ginsenoside Rb1	HFD-induced Kkay mice (C57/BL6)	Gavage, 200 mg/kg/day	Reduced blood glucose, OGTT, serum insulin level, HOMA-IR, liver indexes, increased abundance of *parasitobacteria*, decreased *Alisma* population, unclassified fission of *Prevoteaceae*, stinky bacteria, and anaerobic cell bodies, changed the levels of free fatty acids in fecal metabolites, such as α-linolenic acid, 13-oxydeoxyethylene, oleic acid, 13-hydroxymethyl dehydroepiandroic acid, arachidonic acid, palmitic acid, and stearic acid, and increased the levels of PC (14:0/22:1 (13Z)) and PC (16:0/16:0)	*In vivo*	Zhou et al. (2023)
Ginsenoside Rg1	SD rats	Gavage, 25 mg/kg, 100 mg/kg	Increased the proportion of *Lactobacillus* NK4A136 group and decreased the proportion of *Lactobacillus*	*In vivo*	Peng et al. (2022)
CK	db/db mice	0.03% CK	Reduced the content of intestinal *Bacteroides* and *Parasitobacteria*, increased *Lactobacillus* and *Acetobacter*, reduced intestinal *Bacteroides* and *Parasitobacteria*, increased *Lactobacillus* and *Acetobacter*	*In vivo and* *in vitro*	Chen et al. (2022)
Ginsenoside Rb1	Male Wistar rats	1 g/kg	Restored the disrupted intestinal microflora and increased fecal D-glucosidase activity	*In vivo*	Li et al. (2018)
Ginseng pectin	male SD rats	100 mg/kg and 300 mg/kg	Regulated the composition of GM, activated AMP-activated protein kinase, phosphorylated acetyl-CoA carboxylase, and decreased the expression of sterol regulatory element binding protein-1c and fatty acid synthase	*In vivo*	Ren et al. (2023a)
25(S)-Hydroxyprotopanaxatriol(T19)	HepG2 cells, diabetic mice	100 μg/mL	Reduced the number of bacteria in the intestinal flora, improved the condition of intestinal microflora caused by a high-fat diet/streptozotocin, and increased the relative abundance of *Lactylaceae* bacteria	*In vivo and* *in vitro*	Xu et al. (2020)
GP	Male SD rats	0.27–0.81 g/d	Reduced the downregulation of CYP3A and P-gp expression, affected the distribution of ZO-1, and inhibited the expression of TJ protein	*In vivo and* *in vitro*	Yang et al. (2021)
GP	Male Sprague–Dawley (SD) rats	228 mg/kg/day	Stimulated the growth of Caco-2 cells, facilitated bidirectional transport of Rb1 across monolayers, and increased the PAPP of Rb1 from 10–7 cm/S to 10–6 cm/S	*In vivo*	Shen et al. (2018)
Ginsenoside Rg1	Kunming mice	10–20 mg/kg	Increased the spleen index and the number of T and B cells. In the Rg1 group, the relative abundance of intestinal microflora increased, such as *Alisma*, *Antimycococcaceae*, *Lactococcus*, and Rosaceae, while the potential pathogenic bacteria, such as Spirillaceae, *Dubosiella*, *Mycoplasma*, *Allobacterium*, and *Allobaculum*, decreased significantly	*In vivo*	Yousuf et al. (2022)
Rh4	C57BL/6 mice	20–60 mg/kg	Rh4 significantly suppressed the TLR4-MyD88-MAPK signal pathway. Rh4 treatment significantly increased the levels of short-chain fatty acids (SCFAs) and bile acids (BAs).	*In vivo*	Bai et al. (2021a)
Rk3	Male C57BL/6 mice	30 mg/kg/day and 60 mg/kg/day	Improving the metabolic disorder of intestinal flora by inhibiting the TLR4/NF-κB signal pathway, significantly reducing the ratio of non-Michter/*Bacteroides*, and inhibiting the inflammatory cascade reaction	*In vivo*	Li et al. (2021)
Rk3	C57BL/6JFandd	20 mg/kg/day and 60 mg/kg/day	Enriching *Bacteroides*, *allobacteria*, and *cyanobacteria*, effectively improving intestinal microflora disorders and significantly reducing the ratio of *Firmicuts*/*Bacteroidetes*, by increasing the expression of tight junction proteins (ZO-1, occludin, and claudin-1), reducing the level of colonic inflammatory cytokines, and inhibiting the excessive production of TNFr-α, IL-1β, and IL-6	*In vivo*	Bai et al. (2021b)
GPs	SD rats	50 mg/kg, 100 mg/kg, 200 mg/kg	Restored mTOR-dependent autophagy. Active autophagy reduces inflammation by inhibiting the release of NF-κB, oxidative stress, and cytokines.	*In vivo*	Wang et al. (2021)
PM	SD rats	200 mg/kg/d, 400 mg/kg/d	Decreased the ratio of mycelium to *Bacteroides* B(F/B) and decreased the levels of serum TC, LDL-C, and IgA	*In vivo*	Zhao et al. (2021)
PE	C57BLKS/J- Leprdb/Leprdb (db/db) mice	10 mg/kg	Reduce obesity by activating brown adipose tissue (BAT) and forming beige fat	*In vivo*	Quan et al. (2020)
PE	SD rats	10 mg/mL	Enriched *Ackermann*, *Bifidobacterium, Bacteroides*, and *Proteus* and changed the intestinal flora	*In vivo*	Ren et al. (2023b)
PPD	ob/ob mice	10 mg/kg	Enhanced the integrity of the intestinal wall and regulated specific bacteria	*In vivo*	Lv et al. (2022)
Rg1	ICR mice	10 mg/kg and 40 mg/kg	Inhibited the TLR4/NF-κB pathway and altered GM populations	*In vivo*	Xia et al. (2022)
FG	C57BL/6N mice	390 mg/kg/day	Reduced the abundance of *Bacteroides* S24-7 and *Proteus* S24-7, improved the abundance of short-chain fatty acid-producing bacteria such as *Akkermansia* (of *Verrucommicrobia*) and *Allobaculum*, *Luminococcus*, and *Adlercreutzia* (of Actinomycetes)	*In vivo*	Fan et al. (2019)
(20R)-PPD	C57BL/6J mice and ob/ob mice	10 mg/kg	Improved abnormal glucose and lipid metabolism and could enhance the integrity of intestinal walls	*In vivo*	Fang et al. (2023)
(20R)-(PPD)	ob/ob mice and 10 C57BL/6J	10 mg/kg	Improved the integrity of the intestinal wall, downregulated TNF-α, malondialdehyde, and SOD	*In vivo*	Feng et al. (2023)
FG	Male SD rats	0.5 g/kg/d	Reduced the expression of colonic immune factors TLR4 and NF-κB and returned intestinal microflora to normal	*In vivo*	Qu et al. (2021)
FG	Male SD rats	0.5 g/kg/d	Returned intestinal microflora to normal and downregulated TNF-α, IL-1β, IL-6, and IL-10	*In vivo*	Li et al. (2019)
WEG	Male Balb/c mice	100 mg/kg, two times a day	Increased the relative abundance of bacteria, decreased the relative abundance of *Bacteroides*, *Proteus*, and *Actinomycete*s, increased the relative abundance of *Lactobacillus* and *Streptococcu*s, decreased the relative abundance of *Bacteroides*, restored carbohydrate, amino acid, and energy metabolism to normal levels, and promoted the restoration of mucosal structure	*In vivo*	Li et al. (2019)
CK	HGC-27 cells	10 μM, 20 μM, 30 μM, 40 μM, 50 μM, and 60 μM	Downregulates cyclin B1 and Cyclin D1 expression and downregulates the PI3K/AKT/NF-κB pathway	*in vitro*	Wan et al. (2022)
GPs	C57BL/6J mice	200 mg/kg	Altered the GM and *kynurenine*/*tryptophan* ratio, potentiating the antitumor effect of antiprogrammed cell death 1/programmed cell death ligand 1	*In vivo*	Huang et al. (2022)
GP-n and GP-c	C57BL/6 mice	1.17 mg/mL	Anti-melanoma effects, restored the levels of single-chain fatty acids such as acetic acid and butyric acid, and improved the intestinal microflora ecosystem by upregulating the abundance of *Allobaculum* and *Bifidobacterium*	*In vivo*	Xie et al. (2023)
PG	BALB/c nude mice	1.17 g/kg/d	Inhibited tumor growth by regulating fecal metabolites and intestinal microflora	*In vivo*	Hou et al. (2022)
F2			Altered the intestinal microflora of AD mice, enriched the short-chain fatty acid producing flora, increased propionic acid (PA) content in feces and serum of AD mice, was positively correlated with the number of parasitobacter Kim and *Lactobacillus plantarum* in the intestine, and inhibited the inflammatory response of intestine and skin of AD mice through the G-protein coupled receptor/NF-κB pathway	*In vivo*	Li et al. (2024)
Rb1	C57BL/6 mice	50 mg/kg	Increased the protein levels of SIRT1 and SIRT3 at both transcriptional and post-transcriptional levels	*In vivo*	Lei et al. (2022)
Rb1	BALB/c mice	100 mg/kg, 200 mg/kg	Improved the intestinal microflora imbalance, prevented the metabolism of tryptophan derived from intestinal microflora, and decreased the levels of serotonin receptor 1B (5-HTR1B) and serotonin receptor 2A (5-HTR2A)	*In vivo*	Zeng et al. (2023)
WEG	SD rats	20 g/60 kg	Regulated the intestinal microflora and ultimately reshaped the intestinal microbial ecosystem, triggering several molecular and cellular signaling pathways	*In vivo*	Zhou et al. (2021)
GG and FG	Male ICR mice	400 mg/kg/d	Regulated apoptosis-related proteins, the PI3K/AKT/MTOR pathway, and the SIRT1/NF-κB pathway and restored the diversity and structure of intestinal microflora	*In vivo*	Hao et al. (2022)
Probiotic FG	*C. elegans*		Exerted antioxidant and anti-aging effects by reducing the expression of nematode DAF-2mRNA, upregulating the expression of SKN-1 and SOD-3mRNA, and increasing the relative abundance of beneficial bacteria	*In vivo*	Xu et al. (2023)
PG	Wistar rats	1.62 g/kgbw	Enhanced the relative abundance of beneficial bacteria and reduced the relative abundance of harmful bacteria	*In vivo*	Zhang et al. (2023b)
GSLS	BALB/c, ICR (C.B-17/ICR-+/+Jcl), and ICR mice	5 mg/kg/day	Enhanced the interaction of CCR 10-chemokine ligand (CCL) 28, increased the production of PEDV-specific IgA plasma cells in the intestine, and promoted the migration of intestinal IgA plasma cells to the mammary gland (MG)	*In vivo*	Su et al. (2023)
GS, RG, and BG	SD rats	6.64 g/kg/day, 3.32 g/kg/day	Regulated the hypothalamus–pituitary–thyroid axis, regulated the intestinal flora, and improved the decrease of SCFAs	*In vivo*	Liu et al. (2024)
PG	Male Wistar rats	100 mg/kg	Increased the abundance of *Bifidobacterium* and *Lactobacillus* and increased IL-4, IL-10, and IgA	*In vivo*	Sun et al. (2018)

## 5 The role of PG in related complications

### 5.1 Senescence

Aging is a critical global health challenge, characterized by progressive physical decline and heightened susceptibility to age-related pathologies (Fan et al., 2024). Both PG and RG exert anti-aging effects through mechanisms involving inhibition of acetylcholinesterase (AChE) activity and MDA accumulation, coupled with upregulation of SOD and CAT expression. While both phytomedicines modulate GM diversity, RG demonstrates superior efficacy to PG in enriching probiotic taxa (e.g., *Bifidobacterium, Akkermansia*) and suppressing pro-inflammatory bacterial populations under D-galactose (D-Gal)-induced aging conditions. These findings indicate that RG possesses an enhanced capacity to delay senescence via mechanisms including neuroprotection, cognitive preservation, oxidative stress mitigation, and GM remodeling—likely attributable to its increased levels of total polyphenols, rare ginsenosides, and non-starch polysaccharides (Peng et al., 2021).

Metabolic pathways of medicinal plants regulate key endogenous biomarkers, including phenylalanine/tyrosine metabolism, tryptophan metabolism, and purine metabolism. In this context, RS (likely referring to specific plant-derived compounds, e.g., ginsenosides) alleviates the pathogenesis of AZD (presumably Alzheimer’s disease) through multifaceted mechanisms: correcting dysregulated energy metabolism, attenuating neuroinflammation, modulating GM composition, and restoring neurotransmitter balance (Wang et al., 2021).

### 5.2 Anti-inflammation and immune regulation

Ginsenosides exert comprehensive anti-inflammatory effects via multiple mechanisms: suppressing pro-inflammatory cytokine secretion, restoring immune cell homeostasis, modulating GM diversity, and regulating MAPK/NF-κB/NLRP3 inflammasome signaling pathways (Bai et al., 2023). Fermentation broths derived from PG co-cultured with multi-enzyme-coupled probiotics, which are enriched in ginsenosides, polysaccharides, and viable probiotics, significantly enhanced immune function in immunosuppressed mice and restored GM stability. RG-mediated immunomodulation involves NK cell activation and GM remodeling under stress conditions, with NK cell activity correlating with Th1/Th2 cytokine balance and exhibiting significant GM compositional alterations (Jung et al., 2022). The Qisheng Wan formula (QWF) ameliorates Alzheimer’s disease (AZD)-related cognitive deficits and histopathological damage in rats by reducing Aβ_1-42_ deposition and downregulating NF-κB/TNF-α/IL-6 expression, concurrently restoring GM diversity and suppressing inflammation-associated bacterial taxa (Xiong et al., 2022). Co-administration of *Zingiber officinale* and PG in C57BL/6 mice significantly increased probiotic abundance and reduced pathogenic bacterial populations, though overlapping findings suggest potential consolidation of similar results (Wan et al., 2022).

Comparative studies of sulfur-fumigated ginseng (SGP) *versus* non-fumigated ginseng (NGP) revealed diminished immunomodulatory efficacy in SGP, as evidenced by poorer improvements in body weight, immune organ indices, and leukocyte/lymphocyte profiles. Additionally, SGP promoted differential polysaccharide-GM interactions, resulting in increased pathogenic bacterial growth but reduced SCFA production compared to NGP. Fecal microbiota transplantation confirmed the pivotal role of the GM in mediating these disparities in immunomodulatory effects (Zhao et al., 2023). Studies have further demonstrated that Sijunzi decoction (SJZT) restores GM balance and intestinal barrier integrity in DSS-induced colitis, manifested by reduced colonic injury, increased goblet cell counts, MUC2 secretion, and tight junction protein expression. Notably, *Shigella* infection exhibited a strong negative correlation with body weight and colon length but a positive correlation with disease activity index and IL-1β levels (Wu et al., 2023).

### 5.3 Anti-anxiety

RG and its fermented derivative (FRG) exert anti-inflammatory and neuroprotective effects by inhibiting myeloperoxidase (MPO) activity and NF-κB activation in colonic tissues, while reducing the population of NF-κB^+^/CD11c^+^ cells. Plant metabolites of FRG, including ginsenoside Rd and PPT, ameliorate anxiety/depression and colitis via NF-κB-mediated regulation of brain-derived neurotrophic factor (BDNF) expression and GM remodeling (Han et al., 2020).

Systemic metabolic profiling revealed that total ginsenosides from RG significantly modulate HPA axis markers (adrenocorticotropic hormone [ACTH], corticosterone [CORT]) and substance/energy metabolism indices (Na^+^-K^+^-ATPase, cyclooxygenase [COX], neutrophil extracellular traps [NETs] related protein [NCR], and citrate synthase [CS]). In contrast, the polysaccharide fraction of RG (RGPSF) regulates hypothalamus–pituitary–thyroid (HPT) axis hormones (triiodothyronine/thyroxine). GM analysis demonstrated that the oligosaccharide fraction of RG (RGLPF) increases microbial diversity and the relative abundance of beneficial taxa in depressed rats, with WEG specifically increasing *Bacteroides* abundance and significantly impacting SCFA production (Zhang M. et al., 2023).

Banxia Xiexin decoction alleviated depressive-like behaviors and atherosclerosis (AS)-associated parameters in comorbid murine models, concurrently modulating specific bacterial taxa at the genus level (Liao et al., 2023). In UCDF-induced AZD-like behavioral models, co-administration of FRG and RG attenuated IL-6 expression in the hippocampus and hypothalamus, increased serum corticosterone levels, partially reversed GM dysbiosis, and mitigated colonic inflammatory pathology (Shin et al., 2023).

Evidence demonstrated that PG and *Zizyphus jujuba* seed extract (GS) increased the relative abundance of beneficial bacterial taxa (e.g., *Bacteroides*) while suppressing pathogenic bacterial taxa (e.g., *Proteus and Actinobacillus*). Additionally, GS further regulated glucose and amino acid metabolism, collectively restoring GM structure and metabolic homeostasis in depressed rats (Li et al., 2020; Oh S.-J. et al., 2015).

### 5.4 Other complications

Bu Zang Tong Luo decoction (BZTLD) alleviated hindlimb ischemia in T2DM rats by restoring blood flow and capillary density, concurrently modulating GM composition characterized by increased abundances of beneficial bacteria and suppressed pathogenic taxa. Spearman correlation analysis further revealed significant associations between GM compositional changes and parameters of vascular endothelial growth factor (VEGF) signaling and glucose metabolism (Zheng et al., 2020).

The herbal pair Shenlian (SL, composed of *Coptis chinensis* and PG) reversed the expansion of GM diversity and richness in db/db mice, with principal coordinate analysis (PCoA) confirming significant compositional shifts. The hypoglycemic effect of SL may arise from supertonic and acidic *Bacillus*-mediated regulation of GM, positioning it as a promising candidate for the management of diabetes and obesity (Sun et al., 2022).

Ren Shen Bai Zhu capsule (SZC) exhibited multifaceted therapeutic effects in mice with chronic idiopathic diarrhea (CID), as evidenced by improved body weight, thymus and spleen indices, and colonic histopathological outcomes. GM analysis revealed restoration of microbial structure and suppression of diarrhea-associated bacterial taxa at the genus level, confirming its GM-dependent anti-diarrheal mechanism (Wang et al., 2019). Additionally, PG intervention modulated postoperative metabolites and GM, indicating potential therapeutic advantages for colon cancer recovery (Hanada et al., 2021).

The RG formulations alleviated chronic inflammation and insulin resistance while enhancing glycemic control in T2DM rats (Jiang et al., 2022). Ginseng Dingzhi decoction (GN) modulated GM composition by enriching SCFA-producing bacteria and suppressing pathogenic bacterial taxa, thereby mitigating myocardial injury through the preservation of mitochondrial redox homeostasis under hyperglycemic conditions. Additionally, GN markedly improved cardiac function parameters (e.g., heart failure severity and cardiac hypertrophy) in models of diabetic cardiomyopathy (Wang et al., 2022).

Shengmai turmeric powder (SM) enhanced the efficacy of radiotherapy in nude mice by preserving GM diversity and partially restoring irradiation-induced gut microbiota dysbiosis. Additionally, SM improved cardiac function parameters, including ejection fraction (EF%) and fractional shortening (FS%), reduced myocardial fibrosis, regulated lipid and energy metabolism, and downregulated hypertrophy marker genes. These findings are correlated with gut microbiota compositional alterations observed in isoproterenol-induced cardiac hypertrophy models (Kang et al., 2024; Ming et al., 2022).

Despite its promise, current research on Panax ginseng (PG) faces critical limitations: 1) heavy reliance on animal models (e.g., db/db mice, DSS-colitis models) with poor translatability to humans due to interspecies differences in gut microbiota composition; 2) unaddressed variability across animal strains (e.g., SD rats vs C57BL/6); and human populations; 3) unvalidated anticancer claims (e.g., for Rg3/Rh2) lacking robust clinical evidence; 4) methodological inconsistencies in extract preparation protocols and reported ratios of active metabolites; and 5) mechanistic gaps, including missing strain-level analyses of gut microbiota and detailed insights into host-microbiome interactions.

Addressing these challenges requires standardized clinical trials, rigorous quality control measures, strain-specific mechanistic investigations, and personalized microbiota-based therapies. Integrating these approaches will bridge traditional knowledge with evidence-based medicine, advancing the clinical application of PG and its derivatives.

**FIGURE 7:**
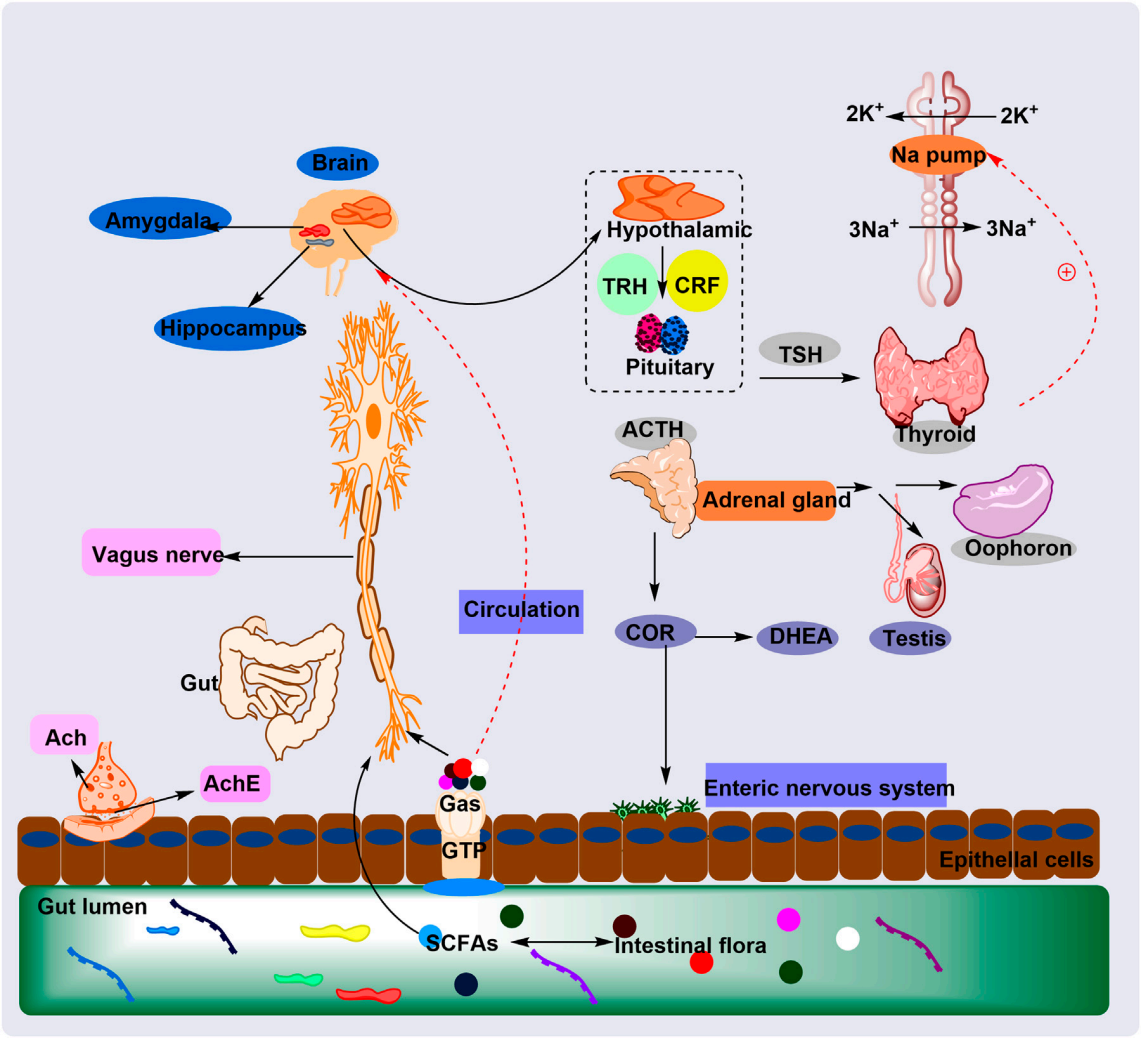
The plant metabolites of PG regulate the HPA axis through the GM to treat cognitive impairment and anxiety mechanisms. The plant metabolites of PG regulate the intestinal microbiota (GM), which in turn regulates the HPA axis and relieves cognitive impairment and anxiety. This occurs through GM-mediated neuroinflammation and normalization of cortisol levels and electrical signals.

Pharmacological activities of PG-related prescriptions are summarized in Table 2, and the brain–gut axis regulatory mechanisms of PG are illustrated in Figure 7.

**TABLE 2:** The role of PG in related complications.

metabolite(s)/Extract(s)	Models	Dose	Detailed activities/mechanisms of action	Application	References
PG and RG	Male ICR mice	500 mg/kg/day	Prevented the increase in acetylcholinesterase (AChE) and malondialdehyde (MDA), increased the expression of superoxide dismutase (SOD) and catalase (CAT), inhibited D-galactose-induced NF-κB translocation and AKT3 and PI3K/AKT pathway activation, delayed the aging process induced by D-Gal, and regulated the diversity of intestinal microorganisms	*In vivo*	Peng et al. (2021)
Ginseng-*Schisandra chinensis*	AD rats	2.582 g/kg	Regulated abnormal energy metabolism, reduced inflammation, and regulated intestinal microflora and neurotransmitters	*In vivo*	Li et al. (2020), Oh et al. (2015b)
AP	Kunming mice	0.01 mL/g/d, i.g	Reduced weight loss and diarrhea, decreased the thymus and spleen index and pathological changes in the ileum and colon, and reduced intestinal inflammatory cytokines (tumor necrosis factor-α, interferon-γ, IL-6, IL-1β, and IL-17)	*In vivo*	Wang et al. (2019)
Ginsenosides	Female BALB/c mice	43.20 mg/mL to 71.00 mg/mL	Improved the immune function of immunosuppressive mice and restored the stability of the intestinal flora	*In vivo*	Bai et al. (2023)
RG	Male C57BL/6N + RAW264.7 cell	300 mg/kg	Regulated intestinal microflora, regulated expression level of Th1/Th2	*In vivo* and *in vitro*	Jung et al. (2022)
Qisheng Wan formula	SD rats	0.00046 g/kg	Ameliorated the cognition processes and histopathological damages due to atopic dermatitis (AD) in rats by decreasing the deposition of Aβ_1-42_ and downregulating the expression of NF-κB, TNF-α, and IL-6 and modulated changes in the diversity and composition of intestinal microbiota to suppress the relative abundance of inflammation-associated microbiota	*In vivo*	Xiong et al. (2022)
*Zingiber officinale* and PG	Male C57BL/6 mice	0.455 g/kg/d	Increased the relative abundance of probiotics, decreased the relative abundance of pathogens, and regulated the intestinal microorganism–metabolite axis	*In vivo*	Wan et al. (2022)
sulfur-fumigated ginseng	BALB/c male mice	80 mg/kg d	Increased the relative abundance of *Firmicute*s, decreased the abundance of *Proteus* and *Actinomycetes,* and reduced the relative abundance of harmful bacteria, such as *Enterococci* and *Shigella*, in immunocompromised mice	*In vivo*	Fang et al. (2023)
SGP, NGP	male ICR mice	9 g/d	Changed the interaction between polysaccharides and intestinal microflora	*In vivo*	Fang et al. (2023)
PG and *Fructus Mume*	SD rats	0.7 g/kg	Improved the damage to the intestinal barrier and increased levels of Muc2 and TJ proteins such as occludin, zonula occludens-1, and claudin-1, inhibited PI3K/Akt/NF-κB signal pathway, and regulated the structure of intestinal microflora	*In vivo*	Zhao et al. (2021)
SJZT	Male C57BL/6J mice	10 g/kg	Reduced colonic tissue injury, increased goblet cell count, MUC2 secretion, and tight junction protein expression, improved the intestinal barrier, and reduced the number of *Proteus* and *Shigella*	*In vivo*	Zhao et al. (2021)
FRG	C57BL/6 mice	10 mg/kg/day, 25 mg/kg/day, 50 mg/kg/day	Reduced stress-induced anxiety/depression-like behaviors, inhibited stress-induced NF-κB activation and NF-κB+/IBA1+ cell count, and reduced anxiety/depression and colitis by modulating NF-κB-mediated expression of brain-derived neurotrophic factors and dysregulation of intestinal flora	*In vivo*	Han et al. (2020)
Total saponins in RG	SD rats	3.24 g/kg and 6.48 g/kg	Reduced blood corticosterone levels, increased the relative abundance of lactic acid bacteria and decreased the relative abundance of *Ackermann protozoa* in depression model rats, increased the diversity and relative abundance of intestinal microflora, and had a strong impact on SCFAs	*In vivo*	Zhang et al. (2023a)
Banxia Xiexin decoction	ApoE−/− mice	0.45 g/kg, 1.35 g/kg, and 4.05 g/kg	Decreased the abundance of *Proteus* and *deferribacterium*; decreased the abundance of *Clostridium*, *Helicobacter*, *Pseudoflavobacter*, acetic factor, *Oscillibacter*, and had abundant glycerol phospholipid metabolism	*In vivo*	Liao et al. (2023)
FRG or RG	C57BL/6 male mice	—	Reduced IL-6 expression in the hippocampus and hypothalamus and increased blood corticosterone levels	*In vivo*	Shin et al. (2023)
PG and the seed of *Zizyphus jujuba* var. spinosa	Wistar rats	0.4 mg/mL	Increased the relative abundance of *Lactobacillus* and *bifidobacterium* but decreased the relative abundance of *streptococcus*, *Shigella*, *Welotella*, and *enterococcus*	*In vivo*	Li et al. (2020)
20(S)-PPD, 20(S)-(PPT)	Male ICR mice	10 mg/kg	Inhibited the levels of serum corticosterone and interleukin-6 induced by stress in immobilized mice	*In vivo*	Oh et al. (2015b)
BZTLF	C57BL/6J mice	5 g (raw materials)/kg/d	Reversed the protein expression changes of vascular endothelial growth factor signaling molecules, decreased vascular endothelial growth factor and hypoxia-inducible factor-1α, increased NO, eNOS and p-ERK1/2, increased the number of beneficial bacteria (*Akkermansia*, *Bifidobacterium,* and *Bacterioidetes*), and decreased the number of harmful bacteria (*Brucella*, *Varicellalla*, *Shigella 28*, and *Kurchia*)	*In vivo*	Zheng et al. (2020)
Shenlian (SL) (*Coptis chinensis* and *Panax ginseng*)	male C57BL/K db/db mice	0.455 g/kg	Reduced the number of Prevotellaceae and *Helicobacteriaceae* bacteria related to lipopolysaccharide biosynthesis, riboflavin metabolism, and peroxisome, increased the abundance of *bacteroides* bacteria, increased the metabolism of starch and sucrose, facilitated the mutual conversion of pentose glucuronic acid, increased the relative abundance of acidibacteroides, and is negatively correlated with fasting blood glucose	*In vivo*	Sun et al. (2022)
Ginseng Dingzhi decoction	C57BL/J mice, HL-1 cell line	30 g/kg/d	Altered the microbial population, increased the proportion of short-chain fatty acids, and reduced the proportion of conditioned pathogenic bacteria to a diabetic phenotype	*In vivo* and *in vitro*	Wang et al. (2022)
SM	BALB/c-nu nude mice	4.29 g/kg	Protected the diversity of colonies and restored the imbalance caused by irradiation	*In vivo*	Kang et al. (2024)
SM	SD rats	3.26 g/kg	The ejection fraction (EF%) and left ventricular shortening rate (FS%) were increased; the relative abundances of Prevotella 9, Lactobacillus, and Clostridium were enhanced; the expression of proteins related to the HIF1α/PPAR signaling pathway was inhibited	*In vivo*	Ming et al. (2022)

## 6 Toxicity and safety research

The safety of dietary supplements and natural products has garnered substantial attention from clinicians and consumers. Given that medicine-food botanical drug substances are generally consumed in higher quantities and with greater frequency than conventional botanical drugs, their safety demands heightened scrutiny from manufacturers, distributors, and end-users (Das et al., 2024). Key exogenous contaminants in these substances encompass pesticide residues, heavy metal accumulations, toxic microorganisms, and sulfites, all of which pose potential health risks when consumed excessively or misused. Improper use of medicine, food, or botanical drug substances may further exacerbate their adverse effects. Consequently, preclinical toxicity assessments are indispensable for PG and PG extracts prior to their integration into medicinal or dietary products. Establishing safety thresholds for toxic by-products and exogenous contaminants is critical to guide population-level intake recommendations.

PG and *Veratrum nigrum* (VN) represent the most clinically critical herb-drug incompatibility pair in TCM, a concept deeply rooted in millennia of empirical clinical practice. In ovariectomized rat models, combined PG-VN administration attenuated the improvements in depression-like behaviors, neurotransmitter balance, and serum estrogen levels compared to monotherapies (PG or VN alone). Metabolomic and GM analyses further revealed that VN co-administration compromised PG’s regulatory effects on eight key metabolic biomarkers and four GM taxa, underscoring the disruptive impact of this incompatible pairing on PG’s pharmacological actions (Lin et al., 2022).

Comparative investigations of PG, RG, and ginseng leaves (GL) have elucidated dose-dependent adverse effects and interactions with the GM. PG demonstrated favorable modulation of the GM by increasing diversity and enriching populations of *Lactobacillus* and *Bifidobacterium*, whereas adverse reactions were primarily dose-dependent. Notably, PG-GL combinations exhibited more severe adverse effects than RG (Ran et al., 2022). Importantly, PG shows minimal toxicity at conventional dosages (3–9 g/day) (Mancuso and Santangelo, 2017), though high-dose intake (>15 g/day) may induce sporadic adverse effects (Siegel, 1980).

In cytotoxicity and repeated-dose toxicity assessments, BG extract displayed no observed adverse effect levels (NOAEL) exceeding 2000 mg/kg body weight in SD rats. Notably, RG extract, ginsenoside Rg5, and 20(S)-Rg3 exhibited comparable safety profiles when tested against six human cancer cell lines (Park et al., 2022). These findings have advanced our mechanistic understanding of TCM therapies and strengthened the rationale for their broader clinical application. The toxicity and safety profiles of PG are summarized in Table 3.

**TABLE 3:** PG-related toxicity and safety research.

metabolite(s)/Extract(s)	Models	Dose	Detailed activities/mechanisms of action	Application	References
*Panax ginseng* (PG) and *Veratrum nigrum* (VN)	Female SD rats	20 mL/kg	Improvement of depression-like behavior, brain neurotransmitters, and decreased serum estrogen levels in ovariectomized rats	*In vivo*	Lin et al. (2022)
PG	Wistar rats	6.64 g/kg/day	Vertical hair disfigurement, nose bleeding, increased rectal temperature, decreased salivary secretion, decreased urine volume, and decreased fecal moisture were observed.	*In vivo*	Ran et al. (2022)
BG extract	A-431 A549 HT-29 NCI-N87 Capan-1 HepG2 Cell lines	1302.1 ± 7.1 μg/mL, 1461.4 ± 22.2 μg/mL, 1228.0 ± 19.9 μg/mL, 1528.6 ± 55.2 μg/mL, 1453.4 ± 20.0 μg/mL, 1357.7 ± 30.3 μg/mL	IC50 was determined from the lowest dose (312.5 μg/mL). BG significantly decreased the survival rate of all cell lines in a dose-dependent manner, except for HT-29 cells, which decreased significantly after a dose of 1250 μg/mL, resulting in complete cell death. Amur431, A549, Capan-1, and HepG2 cells decreased after a dose of 2500 μg/mL, and HT-29 and NCI-N87 cells decreased after a dose of 5000 μg/mL.	*In vitro*	Park et al. (2022)
RG extract	A-431 A549 HT-29 NCI-N87 Capan-1 HepG2 Cell lines	>5000.0 μg/mL	IC50 showed a milder degree of cytotoxic effect than BG only at the high doses. RG extract elicited cytotoxicity in HT-29, NCI-N87, and Capan-1 cells with significant cell death detected from doses of 2500 μg/mL (Capan-1) or doses of 5000 μg/mL (HT-29 and NCI-N87), while A-431 (>1250 μg/mL) and HepG2 (>625 μg/mL) cells dose-dependently responded to medium doses. RG extract caused some cell death in A549 cells.	*In vitro*	Park et al. (2022)
20(S)-Rg3	A-431 A549 HT-29 NCI-N87 Capan-1 HepG2 Cell lines	268.7 ± 7.4 μg/mL, 262.9 ± 5.5 μg/mL, 300.0 ± 5.0 μg/mL, 251.7 ± 1.0 μg/mL, 269.2 ± 5.3 μg/mL, 212.0 ± 20.3 μg/mL	IC50 = 268.7 ± 7.4 262.9 ± 5.5 300.0 ± 5.0 251.7 ± 1.0 269.2 ± 5.3 212.0 ± 20.3 (μg/mL)	*In vitro*	Park et al. (2022)
Rg5	A-431 A549 HT-29 NCI-N87 Capan-1 HepG2 Cell lines	39.2 ± 2.9 μg/mL, 42.8 ± 8.5 μg/mL, 50.0 ± 5.2 μg/mL, 29.4 ± 0.6 μg/mL, 41.6 ± 3.1 μg/mL, 57.9 ± 2.6 μg/mL	IC50 had stronger cytotoxicity	*In vitro*	Park et al. (2022)
Paclitaxel	A-431 A549 HT-29 NCI-N87 Capan-1 HepG2 Cell lines	3.6 ± 0.3 μg/mL, 4.0 ± 1.9 μg/mL, 0.9 ± 0.3 μg/mL, 118.8 ± 53.6 μg/mL, 21.5 ± 5.7 μg/mL, 1.0 ± 0.1 μg/mL	The IC50 values indicated that Paclitaxel had an obvious cytotoxic effect on cancer cells at low doses, and its effect was saturated at doses of more than 13.7–68.3 μg/mL	*In vitro*	Park et al. (2022)
Rb1, Rg1, Rg3, Rg5	SD rats	12.1 mg/g	Caused no abnormal clinical signs or mortality	*In vivo*	Park et al. (2022)

A review of the limitations of included study designs reveals prevalent methodological challenges: small sample sizes (commonly n < 100 per group), lack of blinding procedures, and insufficient randomization methods. For example, while colitis studies reported recovery of β-glucosidase activity, dietary confounders influencing GM composition remained uncontrolled. Most (90%) investigations employed rodent models exhibiting GM compositions significantly divergent from humans (e.g., mice lack key human symbiotic bacterial taxa), and acute disease induction protocols (e.g., DSS-induced colitis) poorly recapitulate the pathogenesis of human chronic diseases. Many studies reported solely on relative GM abundance shifts without functional metabolomic characterization, rendering causal relationships between GM alterations and observed outcomes indiscernible; correlative findings were often erroneously interpreted as causal relationships.

Future research should prioritize addressing three key questions: (1) whether ginsenosides exert effects directly on host cells or indirectly through GM-derived metabolites; (2) how interindividual variability in GM composition modulates treatment responses; and (3) whether long-term use induces adaptive resistance in the GM. Human clinical trials remain scarce (e.g., only 12% of included studies), underscoring the need for large-scale, randomized controlled trials. Sterile mouse models and microbiota transplantation techniques are critical for establishing causal relationships between ginsenosides, GM modulation, and therapeutic outcomes. Standardization of extraction protocols, dosing regimens, and administration methods is urgently required, given the documented 5-fold variability in bioactive phytochemicals across different ginseng preparations.

While ginsenosides demonstrate broad therapeutic potential, their contribution varies by disease context: GM regulation accounts for 30%–50% of metabolic improvements in obesity (Zhao et al., 2021) but only ∼15% of antitumor effects in cancer (Hou et al., 2022). Notably, disease pathologies inherently modulate GM composition, and underreporting of negative findings may artificially inflate reported treatment efficacy, as studies may conflate disease-induced microbiota alterations with direct pharmacological effects.

## 7 Conclusion

This study rigorously evaluated pharmacological parameters across the included literature. (1) Dosage specifications distinguished preclinical (e.g., Rb1 at 10–100 mg/kg in animal models) and clinical (e.g., 200–600 mg/day of ginseng extract) studies, with critical thresholds noted (e.g., Rg1 EC_50_ = 15 μM for anti-inflammation). (2) Model validation confirmed that *in vitro* (Caco-2 cell assay) and *in vivo* (DSS-colitis mouse) systems mirrored human disease mechanisms (e.g., consistency in the TLR4/NF-κB pathway). (3) Over 90% of the results from included studies were positive, with no negative findings for comparison, which weakened the robustness of conclusions. (4) Material standardization ensured taxonomic accuracy (*Panax ginseng* C.A. Mey.) and extraction protocols (70% ethanol, 48 h). Limitations analysis highlighted gaps in dose translation (animal-to-human), long-term safety (>6 months), and microbiota–host interaction mechanisms (e.g., SCFA receptors), proposing future directions: cross-species pharmacodynamic modeling, multicenter trials, and “microbiota–metabolite–host” network analysis.

The GM, often termed the “third organ,” encompasses hundreds of microbial species with a biomass comparable to human somatic cells (Sender et al., 2016). This complex ecosystem, comprising trillions of microbes primarily residing in the gastrointestinal tract, has co-evolved with the host in a mutually beneficial symbiosis. Emerging evidence underscores the pivotal role of GM–botanical drug interactions in modulating mental health, where plant metabolites exert neuroprotective effects through multitarget mechanisms involving both host physiological pathways and microbial community dynamics. Polyphenols and polysaccharides prevalent in herbal medicines demonstrate prebiotic properties that shape GM composition, providing a mechanistic framework to elucidate botanical drug efficacy via the microbiome–gut–brain axis. This paradigm not only advances understanding of traditional plant-based therapies but also facilitates the discovery of novel bioactive metabolites. Notably, drug candidates exhibiting low bioactivity or bioavailability should not be prematurely dismissed, as GM-mediated metabolic transformation may generate therapeutically active metabolites.

Integrating GM modulation into probiotic development strategies holds potential to enhance drug synergy, while interindividual variations in GM composition driven by lifestyle and dietary factors necessitate personalized dose optimization. Leveraging the medicinal-edible duality of PG offers significant potential for developing daily health products. However, comprehensive studies are required to evaluate synergistic effects between PG active plant metabolites and antidepressants/anticancer drugs, mandating rigorous investigation into pharmacokinetic interactions and safety profiles. Future research should prioritize elucidating the bidirectional communication between PG-derived metabolites and GM, while establishing standardized frameworks for quality control and clinical translation. PG’s low toxicity profile positions it as a promising candidate for novel antiviral drug development, provided that systematic toxicity and efficacy evaluations are conducted to support its therapeutic applications.

This perspective highlights the necessity of strengthening basic and clinical research to establish standardized systems for PG application, ensuring its safe and effective integration into modern medical practices. The convergence of traditional knowledge and modern pharmacological approaches may pave the way for innovative health solutions harnessing the therapeutic potential of PG and its interactions with the GM.

Existing studies have demonstrated that ginseng improves metabolic diseases by regulating the gut microbiota; however, the current evidence base is constrained by notable limitations. First, animal model bias is prevalent: 90% of studies rely on rodents (e.g., SD rats, C57BL/6 mice), whose gut microbiota composition significantly diverges from humans, potentially limiting the generalizability of findings to human populations. Second, deficiencies in research design undermine reliability: most clinical studies have small sample sizes (n < 100), lack randomized controlled trials (RCTs), and fail to standardize ginseng dosage (ranging from 100 mg to 3 g/day), weakening the robustness of conclusions. Third, incomplete mechanistic exploration characterizes the field: only 30% of studies integrate metagenomics and metabolomics, with most focusing solely on shifts in bacterial abundance rather than elucidating specific metabolic substance–host interaction mechanisms.

Despite these limitations, current evidence supports two key conclusions: 1) PG polysaccharides significantly increase the abundance of probiotics such as *Akkermansia*; and 2) ginsenoside Rg1 may mitigate intestinal inflammation by inhibiting the NF-κB pathway. Nevertheless, critical gaps remain. For instance, it is unclear whether the microbiota-regulatory effects of ginseng vary across its anatomical parts (roots, stems, leaves), as only three studies have compared these components. Additionally, long-term safety data—particularly regarding the impact of prolonged ginseng use on intestinal barrier function—are lacking. Addressing these challenges will require expanded clinical trials with standardized protocols, multi-omic mechanistic investigations, and comparative studies of ginseng’s anatomical components to advance our understanding of its gut microbiota-dependent therapeutic potential.

In summary, this review underscores the significant translational potential of PG in personalized medicine, primarily driven by its interaction with the GM. PG modulates GM composition, increasing the bioconversion of ginsenosides (e.g., Rb1, Rg3) into active metabolites such as CK, which directly influences therapeutic efficacy. Baseline GM profiling (e.g., abundance of *Bifidobacterium* and *Lactobacillus*) can predict individual responses, enabling biomarker-guided treatment stratification. Future research should prioritize addressing the following key issues: Standardized research design: Conduct multicenter RCTs with clear definitions of ginseng dosage, treatment duration, and standardized extracts (e.g., total ginsenosides ≥50%). Multi-omics integrated analysis: Combine metagenomics, metabolomics, and transcriptomics to unravel the molecular network underpinning the “microbiota–metabolites–host immunity” axis. Mechanistic deepening: Verify causal relationships through fecal microbiota transplantation or germ-free mouse models.

By integrating microbiota analysis into PG therapy, this approach could enhance precision medicine, reduce interindividual variability, and advance PG from traditional empirical use to evidence-based prevention and treatment of chronic diseases.

## Glossary

AAD: antibiotic-associated diarrhea
AChE: acetylcholinesterase
AD: atopic dermatitis
AGM: combination of freeze-dried aronia
AKI: acute kidney injury
AMO: *Atractylodes macrocephala*
AMP: adenosine monophosphate
AS: atherosclerosis
ASD: Alzheimer’s disease
BG: black ginseng
BHRS: Baihu Renshen decoction
BZTLD: Bu Zang Tong Luo decoction
CAT: catalase
CK: compound K
DJZT: Dajianzhong decoction
DSS: dextran sulfate sodium salt
FAD: fatty acid diet
FG: fermented ginseng
FRG: fermented red ginseng
FST: forced swimming
GABAA: gamma-aminobutyrate A
GG: garden ginseng
GF: ginseng under forest
GL: ginseng leaves
GM: gut microbiota
GN: Ginseng Dingzhi decoction
GPS: Ginseng pectin
GSLS: ginsenosides from stems and leaves
HPA: hypothalamus–pituitary–adrenal
HPLC-TQ-MS: high-performance liquid chromatography coupled with triple quadrupole mass spectrometry
IBD: inflammatory bowel disease
IG: immunoglobulin
IL: interleukin
LY: Liangyi paste
MA: myristic acid
MDA: malondialdehyde
MG: mammary gland
MGBA: microbiome–gut–brain axis
NGP: non-fumigated ginseng
PD-1/PD-L1: potentiating the antitumor effect of antiprogrammed cell death 1/programmed cell death ligand 1
PE: ginseng extract
PF: *Panax ginseng* and *Fructus Mume*
PG: *Panax ginseng* C. A. Mey.
PPD: protopanaxadiol, precancerous lesions of gastric cancer
PPS: Peyer’s plaques
PPT: protopanaxatriol
PTP-1B: protein tyrosine phosphatase 1B
QYG: Qiong-Yu-Gao
RG: red ginseng
RS: *Radix ginseng-Schisandra chinensis*
RSGB: Renshen Guben prescription
SCFA: short-chain fatty acid
SGP: sulfur-fumigated ginseng
SJZT: Sijunzi decoction
SL: *Coptis chinensis* and *Panax ginseng*
SOD: superoxide dismutase
STZ: streptozotocin
SZC: RenShenbaizhu capsule
TCM: traditional Chinese medicine
T2DM: type 2 diabetic
TLR4: toll-like receptor 4
TNF-α: tumor necrosis factor-α
TST: tail hanging tasks
UC: ulcerative colitis
UCDF: ulcerative colitis and depression
VN: *Veratrum nigrum*
WEG: water extract of ginseng
WFC: Weifuchun capsule
5-HT: 5-hydroxytryptamine
