# Correction: The potential of medicinal food plant *Panax ginseng* C. A. Mey. in managing chronic diseases via gut microbiota regulation: a systematic review of mechanisms and evidence

**DOI:** 10.3389/fphar.2025.1697027

**Published:** 2025-09-17

**Authors:** Meng Gao, Haijing Wang, Xiaojing Chen, Wensheng Wang, Yongmei Liu

**Affiliations:** ^1^ Neck-Shoulder and Lumbocrural Pain Hospital of Shandong First Medical University, Shandong First Medical University & Shandong Academy of Medical Sciences, Jinan, China; ^2^ Qingdao Traditional Chinese Medicine Hospital, Qingdao Hiser Hospital Affiliated of Qingdao University, Qingdao, China; ^3^ Qingdao Haici Traditional Chinese Medicine Medical Group North Campus(Qingdao Hongdao People’s Hospital), Qingdao, China; ^4^ Institute of Brain Science and Brain-Inspired Research, Shandong First Medical University and Shandong Academy of Medical Sciences, Jinan, Shandong, China

**Keywords:** medicinal food plant, *Panax ginseng* C. A. Mey., gut microbiota, pharmacology, plant metabolites, toxicology

Affiliation “Neck-Shoulder and Lumbocrural Pain Hospital of Shandong First Medical University, Shandong First Medical University & Shandong Academy of Medical Sciences, Jinan, China” was erroneously given as “Shandong First Medical University Affiliated Hospital, Neck, Jinan, China.”

Affiliation “Qingdao Traditional Chinese Medicine Hospital, Qingdao Hiser Hospital Affiliated of Qingdao University” was erroneously given as “Qingdao Hiser Hospital Affiliated of Qingdao University, Qingdao, China.”

Author Wensheng Wang was erroneously spelled as Wengsheng Wang.

The original article has been updated.

